# Exploring the synergistic effects of Life’s Essential 8, insulin resistance, and CRP on cardiometabolic multimorbidity risk

**DOI:** 10.3389/fnut.2025.1598659

**Published:** 2025-07-16

**Authors:** Jiang Liu, Chuang Yang, Wenke Cheng, Daidi Li

**Affiliations:** ^1^Department of Cardiology, Yingtan People’s Hospital, Yingtan, Jiangxi Province, China; ^2^Department of Cardiology, The First Affiliated Hospital of Bengbu Medical University, Bengbu, China; ^3^Department of Nephrology and Medical Intensive Care, Charité-Universitätsmedizin Berlin, Freie Universität Berlin and Humboldt-Universität zu Berlin, Berlin, Germany

**Keywords:** triglyceride glucose, triglyceride glucose-body mass index, triglyceride glucose-waist circumference, triglyceride glucose-waist height ratio, Life’s Essential 8, cardiometabolic multimorbidity

## Abstract

**Background:**

Cardiometabolic multimorbidity (CMM) refers to the co-occurrence of two or more cardiometabolic diseases (CMDs), including coronary artery disease (CAD), stroke, and type 2 diabetes mellitus (T2DM), posing a substantial public health concern. Although Life’s Essential 8 (LE8), a cardiovascular health (CVH) metric incorporating behavioural and metabolic factors, has been developed, its relationship with CMM remains unexplored. This study examines the independent and combined effects of LE8, insulin resistance (IR) and C-reactive protein (CRP) on CMM risk.

**Methods:**

In this prospective cohort study, 304,568 UK Biobank participants without CMM at baseline were followed. The association between LE8 and CMM risk was assessed using Cox proportional hazards models, and dose–response relationships were evaluated using restricted cubic splines. Mediation analyses were conducted to determine the roles of triglyceride-glucose index (TyG, an IR indicator) and CRP in mediating the LE8-CMM association.

**Results:**

Over a 14.2-year follow-up, 5,441 participants developed CMM. Higher LE8 scores were significantly associated with reduced CMM risk (hazard ratio [HR] per 10-point increase: 0.65; 95% confidence interval [CI]: 0.63–0.67). Accelerated failure time models indicated that increased LE8 scores delayed CMM onset by up to 47.3 months. Mediation analyses showed that TyG and CRP accounted for 18.8 and 2.9% of LE8’s protective effect on CMM, respectively.

**Conclusion:**

Maintaining high LE8 scores is associated with a lower risk of developing CMM, partially mediated by reductions in IR and inflammation. Promoting CVH and addressing metabolic and inflammatory factors may help prevent CMM and reduce its burden on public health.

## Introduction

Cardiometabolic multimorbidity (CMM), defined as the co-occurrence of two or more cardiometabolic diseases (CMDs) including coronary artery disease (CAD), stroke and type 2 diabetes mellitus (T2DM), is a growing global health issue (1). Advances in healthcare have prolonged the survival of patients with a single CMD, but these gains have also increased the risk of additional conditions, leading to a notable rise in CMM prevalence. In the United States, CMM prevalence among middle-aged and elderly adults has increased from 9.4 to 14.1% over the past two decades, while in China, it increased from 2.41% in 2010 to 5.94% in 2016 (2, 3). The co-occurrence of multiple CMDs compounds health risks, with evidence showing that CMDs significantly increase mortality, disability and healthcare burdens beyond the risks associated with each CMD individually (4–6).

Unhealthy lifestyle factors are strongly associated with CMM. Studies report that smoking, alcohol consumption, physical inactivity and obesity significantly increase the risk of CMM (7–9). Moreover, the effects of these lifestyle behaviours are cumulative, with each additional unhealthy behaviour raising the likelihood of developing CMM (10).

In 2022, the American Heart Association (AHA) developed Life’s Essential 8 (LE8), a comprehensive cardiovascular health (CVH) metric, comprising eight key factors: nicotine exposure, physical activity, sleep health, dietary quality, body mass index (BMI), blood pressure, cholesterol levels and blood glucose (11). Higher LE8 scores have been associated with a lower risk of cardiovascular diseases (CVDs), as well as stroke and T2DM (12–14). Additionally, insulin resistance (IR), often linked to obesity and a sedentary lifestyle, contributes to dysregulated glucose metabolism and systemic inflammation, increasing the risks for T2DM and CVDs (15). Previous study identified IR as a significant predictor of CMM (16).

However, despite these associations, the relationship between LE8, IR and CMM remains unexplored, particularly regarding potential mediating roles. To address this gap, we conducted a large-scale cohort study using UK Biobank data to examine the combined effects of LE8 and IR on CMM development. Particularly to explore the potential mediating roles of LE8 and IR in the progression of CMM.

## Methods

### Study population

The UK Biobank is a large prospective cohort study that enrolled over 500,000 participants aged 37 to 73 from England, Scotland and Wales between 2006 and 2010. This study investigates genetic; lifestyle and environmental factors linked to a variety of diseases. Data collection included touchscreen questionnaires, physical measurements and biological samples, supplemented by self-reported medical histories and demographic information. The UK Biobank received ethical approval from the National Health Service (NHS) and the National Research Ethics Service (NRES) (17). This study followed the Strengthening the Reporting of Observational Studies in Epidemiology (STROBE) reporting guideline (Appendix).

### Assessment of LE8 and CVH

The LE8 consists of eight key indicators: diet, physical activity, tobacco/nicotine exposure, sleep health, BMI, blood lipids, blood glucose and blood pressure. Diet score was assessed using nine dietary behaviours collected at baseline, including: (1) fruit and vegetable intake < 5 servings/day; (2) consumption of fish < once per week; (3) processed meat intake > once per week; (4) red meat intake ≤ once per week; (5) use of full-fat milk or rarely/never consuming milk; (6) use of spreads other than low-fat or unsaturated types; (7) cereal intake ≤ 5 bowls/week; (8) frequent addition of salt to food (sometimes, usually, or always) and (9) water intake < 6 glasses/day (18). For each suboptimal dietary behaviour, participants were assigned 1 point; otherwise, 0 points. The total diet score ranged from 0 (healthiest diet) to 9 (least healthy). To integrate with the LE8 scale, the raw score was reversed and rescaled to a 0–100 range, with higher values reflecting better diet quality. Physical activity was assessed by weekly minutes of moderate-to-vigorous physical exercise per week. Baseline questionnaires captured tobacco exposure and sleep health, while BMI was calculated using weight and height measured at enrolment. Blood lipid levels and HbA1c were measured at the UK Biobank central laboratory, and blood pressure was recorded using an Omron device. Each LE8 component was individually scored from 0 to 100 according to the AHA guidelines, with higher scores indicating better CVH (11). The overall LE8 score was calculated as the unweighted mean of the eight metrics, meaning each component contributed equally to the total score. The LE8 scoring details are summarised in Supplementary Table S1.

The LE8 score, ranging from 0 to 100, represents the average of the eight component scores, with higher scores indicating better CVH. CVH levels were categorised as low (LE8 score < 50), moderate (50 ≤ LE8 score < 80) and high (LE8 score ≥ 80), with scores of 80 or higher indicating ideal CVH.

### Assessment of IR and inflammation biomarker

Baseline blood samples from each participant were analysed in a central laboratory for various biochemical indicators, including triglycerides (TG), fasting plasma glucose (FPG) and C-reactive protein (CRP). In this study, the TyG index was used as a surrogate marker for IR and calculated using the formula: TyG = ln [TG (mg/dL) × FPG (mg/dL) / 2] (19). CRP levels were used to assess systemic inflammation.

### Ascertainment of CMM and CMDs

This study included three CMDs: CAD, stroke and T2DM. The primary outcome, CMM, was defined as the presence of two or more CMDs. Secondary outcomes included the individual occurrence of each CMD. Diagnoses were based on the International Classification of Diseases, Tenth Revision (ICD-10) codes: CAD (I20-I25), stroke (I60-I64) and T2DM (E11). Data were obtained from death registries, primary care records, hospital records and participant self-reports. Participants were followed from enrolment until the first CMD occurrence, death, or the end of the observation period (May 1, 2023).

### Assessment of other variables

Baseline assessments of sociodemographic and lifestyle factors were conducted using a touchscreen questionnaire. Variables included age, sex (male, female), ethnicity (White, Others) and socioeconomic status, measured by the Townsend Deprivation Index (TDI). Education levels were classified according to the International Standard Classification of Education (ISCED) Code, ranging from levels 1 to 5 (Supplementary Table S2) (20). Annual household income before tax was categorised into five groups: <£18,000, £18,000–29,999, £30,000–51,999, £52,000–100,000 and >£100,000. Lifestyle behaviours were assessed including physical activity (measured by Metabolic Equivalent of Task [MET] scores) and alcohol consumption, categorised into six frequency groups (‘never’, ‘special occasions only’, ‘one to three times a month’, ‘once or twice a week’, ‘three or four times a week’ and ‘daily or almost daily’). Information on family history of CVD and current use of medications, (e.g., antihypertensives and lipid-lowering drugs) was recorded. Data on annual average concentrations of PM_2.5_, PM_2.5–10_, PM_10_, NO_2_ and NO_x_ were obtained from the UK Department for Environment, Food & Rural Affairs (DEFRA) (21).

### Participant selection criteria

**Figure 1:**
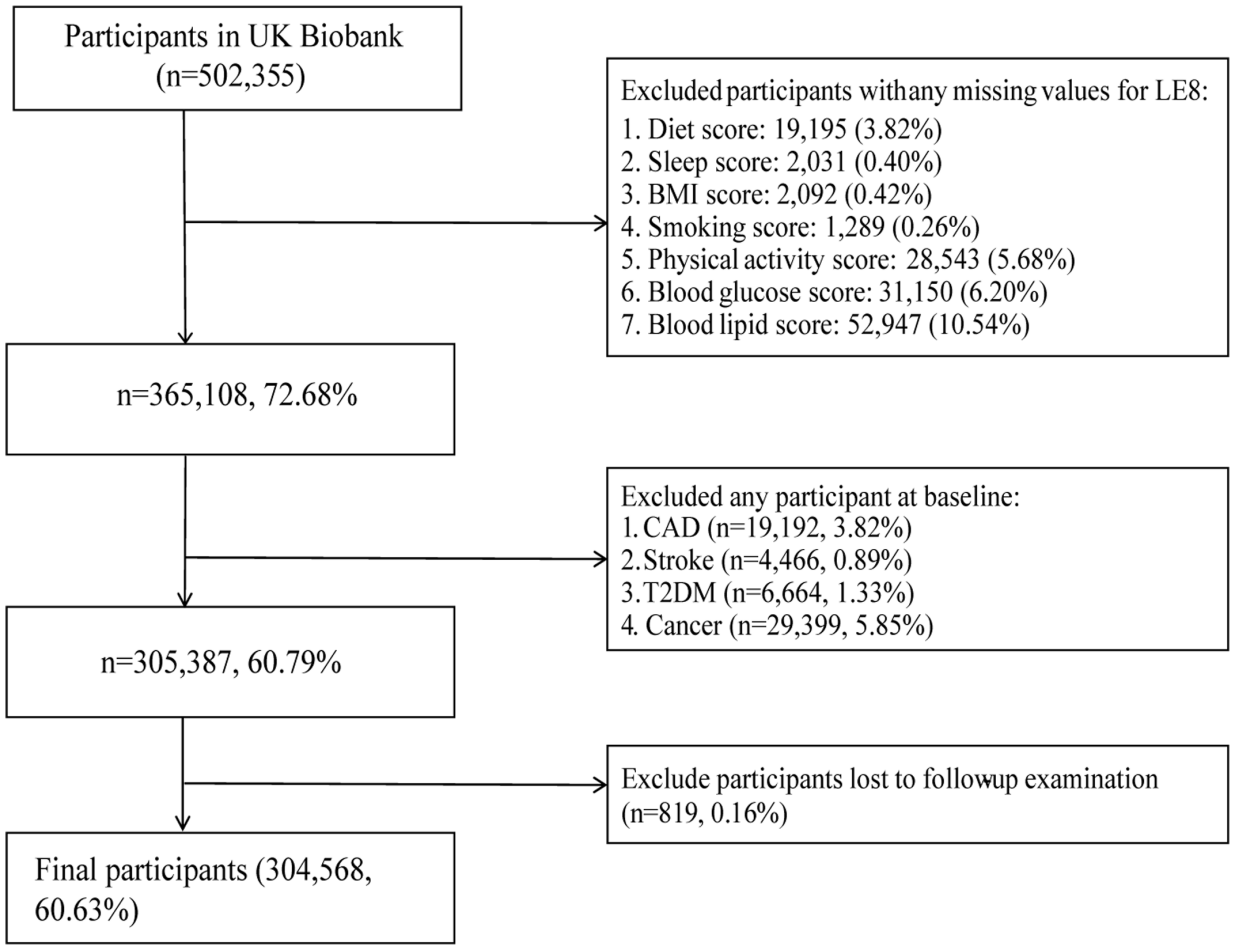
Flowchart depicting the selection of participants.

502,355 participants were recruited into the UK Biobank cohort. Participants with incomplete LE8 data were initially excluded (*n* = 137,247). Additional exclusions included those diagnosed with CAD (*n* = 19,192), stroke (n = 4,466), T2DM (*n* = 6,664), or any cancer (*n* = 29,399) at baseline, as well as participants lost to follow-up (*n* = 819). Finally, a total of 304,568 participants were included in the final analysis (Figure 1).

### Statistical analysis

Baseline covariate missing values were reported in Supplementary Table S3. Missing data were addressed using multiple imputation by chained equations (MICE) with a random forest method. This approach is well-suited to handling mixed data types and non-linear relationships. Five imputed datasets were generated, and one of them was used for the main analysis. Imputation accuracy was assessed using the normalised root mean squared error (NRMSE = 0.069) for continuous variables and the proportion of falsely classified entries (PFC = 0.221) for categorical variables.

Missing data percentages for key baseline variables were as follows: CRP (16%), annual household income (12%), and education level (1%). Other covariates, including race and ethnicity, TDI, medication use, alcohol intake frequency, and TyG index, had minimal or no missingness (<1%). To assess potential selection bias from these exclusions, we compared baseline characteristics between included and excluded participants, and the results were presented in Supplementary Table S4. Baseline variables were categorised by LE8 levels (low, moderate, high); continuous variables were presented as median (standard deviation [SD]); and categorical variables as frequency (percentage). Differences across CVH groups were assessed using ANOVA for continuous variables and chi-square tests for categorical variables. Besides, differences between the study cohort and the general population (the overall UK Biobank population) were also compared. Kaplan–Meier (KM) survival curves were used to assess CMM and CMD risks across CVH groups, with log-rank tests used for group comparisons. Cox proportional hazard models evaluated CMM and CMD risks associated with LE8 scores (per 10-point increase or grouped as low, moderate and high). TyG and CRP data were categorised into quartiles and standardised using z-scores to examine CMM risk per 1 SD increase or by quartiles of TyG and CRP. Proportional hazard assumptions were verified with Schoenfeld residuals and the results were shown in Supplementary Figure S1. Collinearity was assessed using the variance inflation factor (VIF), with VIF < 5 indicating acceptable collinearity for model adjustments. Model 1 was adjusted for age, sex, and ethnicity, while Model 2 included additional adjustments for TDI, education level, income, family CVD history, lipid-lowering and antihypertensive medication use and alcohol intake frequency. Restricted cubic splines (RCS) were used to examine dose–response relationships between LE8 scores and both CMM and CMDs. The association between LE8 scores and CMM risk across TyG and CRP quartiles was also evaluated.

Additionally, to assess combined effects, participants were classified into 12 groups based on different LE8 and TyG or CRP levels. Using the low CVH and high TyG or CRP group as the reference, we evaluated CMM risk across combinations.

To further characterise the temporal dimension of the association between CVH and CMM, we employed an accelerated failure time (AFT) model, which estimates the effect of covariates on the survival time directly, rather than on the hazard function. Unlike Cox proportional hazards models, AFT models do not assume proportionality of hazards and instead provide interpretable estimates of time ratios or median survival time differences (22). This modelling approach allowed us to quantify the extent to which higher CVH levels were associated with delayed onset of CMM.

Subgroup analyses were performed by sex, age, ethnicity, alcohol intake, TDI, family CVD history, antihypertensive and lipid-lowering drug use, as well as TyG and CRP levels. Several sensitivity analyses were conducted to assess the robustness of our findings: (1) Given the older age profile of our cohort, the potential influence of competing risks due to all-cause mortality was explicitly evaluated using Fine-Gray competing risks regression, in line with standard practices from previous UK Biobank studies (23–25). (2) Participants with <2 years of follow-up were excluded. (3) Participants with missing baseline covariates were excluded. (4) Participants who reported prior use of antihypertensive or lipid-lowering medications were excluded. (5) TyG and CRP were excluded from the adjustment set. (6) Additional adjustments were made for ambient particulate pollution indicators (PM_2.5_, PM_2.5–10_, PM_10_, NO_2_, NO_x_) to address potential confounding from pollution exposure. (7) To address the limitation of using a single imputed dataset, pooled analyses based on the remaining four imputed datasets were performed. (8) To strengthen the assessment of IR, we repeated the analysis using three alternative IR-related markers: triglyceride-to-high-density lipoprotein cholesterol ratio (TG/HDL-C), the metabolic score for insulin resistance (METS-IR), and estimated glucose disposal rate (eGDR). All three indices have been validated in large-scale cohorts as surrogate markers for IR or insulin sensitivity, with detailed calculation methods provided in the original studies (26–28). (9) Finally, to confirm the robustness of the LE8 scoring method, we reconstructed the LE8 score using the traditional Dietary Approaches to Stop Hypertension (DASH) diet score. The methodology for constructing the DASH score has been described in a previously published study (29).

### Explorative analyses

Receiver operating characteristic (ROC) curves assessed the predictive accuracy of the CVH score for CMM development. Mediation analysis was conducted to determine if TyG or CRP mediated the association between CVH and CMM risk. In this analysis, CVH was treated as the exposure, TyG or CRP as the mediator and CMM as the outcome, with significance tested using 500 bootstrap samples (30).

All statistical analyses were performed using R software (version 4.3.1), with a two-tailed *p*-value < 0.05 considered statistically significant.

## Results

### Characteristics of baseline population

Among the 304,568 participants with complete LE8 scores, the mean age was 55.75 ± 8.09 years, with 54.07% male and 95.37% identifying as Caucasian (Table 1). Baseline characteristics across different CVH groups are detailed in Table 1. LE8 indicator levels increased progressively from the low to high CVH groups, while TyG and CRP levels decreased correspondingly. In addition, compared with the overall UK Biobank participants, the included analytic cohort showed generally similar baseline characteristics, with some moderate differences observed in income (SMD = 0.26), lipid-lowering drug use (SMD = 0.37), and antihypertensive use (SMD = 0.29) (Supplementary Table S5).

**Table 1:** Baseline demographic and clinical characteristics across CVH groups.

Characteristics	Total	Low	Moderate	High	*p*
*N*	304,568	27,596	227,887	49,085	
Age, years	55.75 ± 8.09	56.87 ± 7.63	56.26 ± 7.99	52.74 ± 8.14	<0.001
Male	164,689 (54.07%)	16,590 (60.12%)	109,622 (48.10%)	13,667 (27.84%)	<0.001
White	290,477 (95.37%)	26,270 (95.19%)	217,342 (95.37%)	46,865 (95.48%)	0.203
Townsend deprivation index	−1.47 ± 2.99	−0.61 ± 3.33	−1.50 ± 2.97	−1.79 ± 2.79	<0.001
Education levels					
CSEs or equivalent	43,384 (14.24%)	7,085 (25.67%)	33,101 (14.53%)	3,198 (6.52%)	<0.001
A levels/AS levels or equivalent	82,625 (27.13%)	8,064 (29.22%)	62,807 (27.56%)	11,754 (23.95%)	
Other professional qualification	36,156 (11.87%)	2,882 (10.44%)	26,825 (11.77%)	6,449 (13.14%)	
College or University degree	15,367 (5.05%)	1,431 (5.19%)	11,782 (5.17%)	2,154 (4.39%)	
None of the above	127,036 (41.71%)	8,134 (29.48%)	93,372 (40.97%)	25,530 (52.01%)	
Annual household income before tax, £					<0.001
<18,000	62,169 (20.41%)	8,410 (30.48%)	47,067 (20.65%)	6,692 (13.63%)	
18,000–30,999	75,734 (24.87%)	7,032 (25.48%)	58,253 (25.56%)	10,449 (21.29%)	
31,000–51,999	82,282 (27.02%)	6,763 (24.51%)	61,517 (26.99%)	14,002 (28.53%)	
52,000–100,000	66,486 (21.83%)	4,501 (16.31%)	48,471 (21.27%)	13,514 (27.53%)	
>100,000	17,897 (5.88%)	890 (3.23%)	12,579 (5.52%)	4,428 (9.02%)	
History of heart diseases family	118,789 (39.0%)	10,883 (39.44%)	89,931 (39.46%)	17,975 (36.62%)	<0.001
Lipid-lowering drugs	35,769 (11.74%)	5,296 (19.19%)	27,394 (12.02%)	3,079 (6.27%)	<0.001
Antihypertensives	49,353 (16.2%)	7,896 (28.61%)	38,663 (16.97%)	2,794 (5.69%)	<0.001
Alcohol intake frequency					<0.001
Never	21,183 (6.96%)	2,234 (8.10%)	15,389 (6.75%)	3,560 (7.25%)	
Special occasions only	31,841 (10.45%)	3,492 (12.65%)	23,224 (10.19%)	5,125 (10.44%)	
One to three times a month	33,640 (11.05%)	3,043 (11.03%)	24,670 (10.83%)	5,927 (12.07%)	
Once or twice a week	79,649 (26.15%)	6,550 (23.74%)	58,775 (25.79%)	14,324 (29.18%)	
Three or four times a week	73,936 (24.28%)	5,408 (19.60%)	55,839 (24.50%)	12,689 (25.85%)	
Daily or almost daily	64,319 (21.12%)	6,869 (24.89%)	49,990 (21.94%)	7,460 (15.20%)	
TyG index	8.69 ± 0.56	9.10 ± 0.56	8.72 ± 0.53	8.30 ± 0.45	<0.001
CRP (mmol/l)	2.46 ± 4.12	4.24 ± 5.29	2.45 ± 4.03	1.53 ± 3.45	<0.001
Total LE8 score	67.10 ± 12.56	43.22 ± 5.53	66.08 ± 7.88	85.25 ± 4.36	<0.001
Diet score	47.31 ± 31.36	21.75 ± 25.41	45.77 ± 30.51	68.83 ± 24.01	<0.001
Blood pressure score	43.87 ± 31.77	23.22 ± 22.75	40.08 ± 29.47	73.10 ± 27.89	<0.001
Blood glucose score	93.06 ± 16.67	80.25 ± 24.17	93.38 ± 16.21	98.76 ± 7.17	<0.001
Sleep health score	89.69 ± 18.23	78.47 ± 24.92	89.79 ± 17.84	95.56 ± 11.42	<0.001
Body mass index score	69.90 ± 28.03	42.21 ± 27.62	68.60 ± 26.71	91.49 ± 15.06	<0.001
Physical activity score	83.03 ± 34.92	38.62 ± 46.20	85.25 ± 32.50	97.72 ± 10.76	<0.001
Tobacco/nicotine exposure score	62.17 ± 36.60	30.48 ± 32.51	60.99 ± 36.12	85.48 ± 23.30	<0.001
Blood lipid score	47.75 ± 29.64	30.73 ± 25.87	44.78 ± 27.77	71.09 ± 27.30	<0.001

CSE, Certificate of Secondary Education; AS levels, Advanced Subsidiary Level Education; TyG, triglyceride-glucose; CRP, C-reactive protein; LE8, Life’s Essential 8; Low CVH, LE8 score < 50 points. Moderate CVH, LE8 score 50–80 points. High CVH, LE8 score ≥ 80 points.

### Association of LE8 with the risk of CMM and specific CMDs

Over a mean follow-up of 14.2 years, 5,441 cases of CMM were identified (Table 2). Incidence rates of CMM and CMDs decreased across CVH groups from low to moderate to high (Table 2). After adjusting for age, sex and ethnicity, higher LE8 levels were significantly associated with reduced risks of CMM, CAD, stroke and T2DM (all *P* for trend < 0.01; Table 2). The inverse association between LE8 score and CMD risks remained robust after additional adjustments in Model 2 (all *P* for trend < 0.001). Each 10-point increase in LE8 score was linked to a lower risk of CMM, CAD, stroke and T2DM, with HRs of 0.65, 0.81, 0.85 and 0.63, respectively (Table 2). KM curves further demonstrated a decreasing trend in CMM and CMD risk with higher CVH levels (all *P* for log-rank test < 0.05, Supplementary Figure S2).

**Table 2:** The association between LE8 score and the risk of CMM and specific CMDs.

Type	Cases	Incidence^a^	Model 1		Model 2	
			HR (95%CI)	*p*	HR (95%CI)	*p*
CMM	5,441	1.29				
Low	1,721	4.68	Reference		Reference	
Moderate	3,589	1.14	0.26 (0.25–0.28)	<0.001	0.44 (0.41–0.47)	<0.001
High	131	0.19	0.06 (0.05–0.08)	<0.001	0.17 (0.14–0.2)	<0.001
*P* for trend			<0.001		<0.001	
Per 10-point increase			0.53 (0.52–0.54)	<0.001	0.65 (0.63–0.66)	<0.001
CAD	23,570	4.98				
Low	4,089	11.63	Reference		Reference	
Moderate	17,972	5.86	0.56 (0.54–0.57)	<0.001	0.69 (0.67–0.71)	<0.001
High	1,509	2.21	0.3 (0.28–0.31)	<0.001	0.43 (0.41–0.46)	<0.001
*P* for trend			<0.001		<0.001	
Per 10-point increase			0.75 (0.74–0.76)	<0.001	0.81 (0.8–0.82)	<0.001
Stroke	7,228	1.72				
Low	1,093	2.87	Reference		Reference	
Moderate	5,528	1.76	0.64 (0.6–0.68)	<0.001	0.72 (0.68–0.77)	<0.001
High	607	0.88	0.45 (0.41–0.5)	<0.001	0.55 (0.5–0.62)	<0.001
*P* for trend			<0.001		<0.001	
Per 10-point increase			0.81 (0.8–0.83)	<0.001	0.85 (0.83–0.86)	<0.001
T2DM	17,668	4.28				
Low	5,470	15.73	Reference		Reference	
Moderate	11,856	3.83	0.25 (0.2–0.26)	<0.001	0.44 (0.43–0.46)	<0.001
High	342	0.5	0.04 (0.04–0.04)	<0.001	0.12 (0.11–0.14)	<0.001
*P* for trend			<0.001		<0.001	
Per 10-point increase			0.5 (0.49–0.51)	<0.001	0.63 (0.63–0.64)	<0.001

CVH, Cardiovascular health; CMM, cardiometabolic multimorbidity; CMDs, cardiometabolic diseases; CAD, coronary artery disease; T2DM, type 2 diabetes mellitus; HR, hazard ratio; CI, confidence interval. Model 1 adjusted age, sex, and race. Model 2 was further adjusted for Townsend deprivation index, education levels, annual household income, family history of heart diseases, history use of lipid lowering drugs, antihypertensives, alcohol intake frequency, TyG index and CRP levels. ^a^1,000 person-year.

### Associations of TyG and CRP with the risk of CMM

After progressively adjusting for confounders, higher quartiles of TyG and CRP were associated with an elevated risk of CMM (all *P* for trend < 0.001). Specifically, each SD increase in TyG or CRP was linked to a 39% (HR: 1.39, 95% CI: 1.35–1.43) and 12% (HR: 1.12, 95% CI: 1.10–1.14) higher risk of CMM, respectively (Supplementary Table S6).

### Dose-dependent relationship of LE8 score and CMM

**Figure 2:**
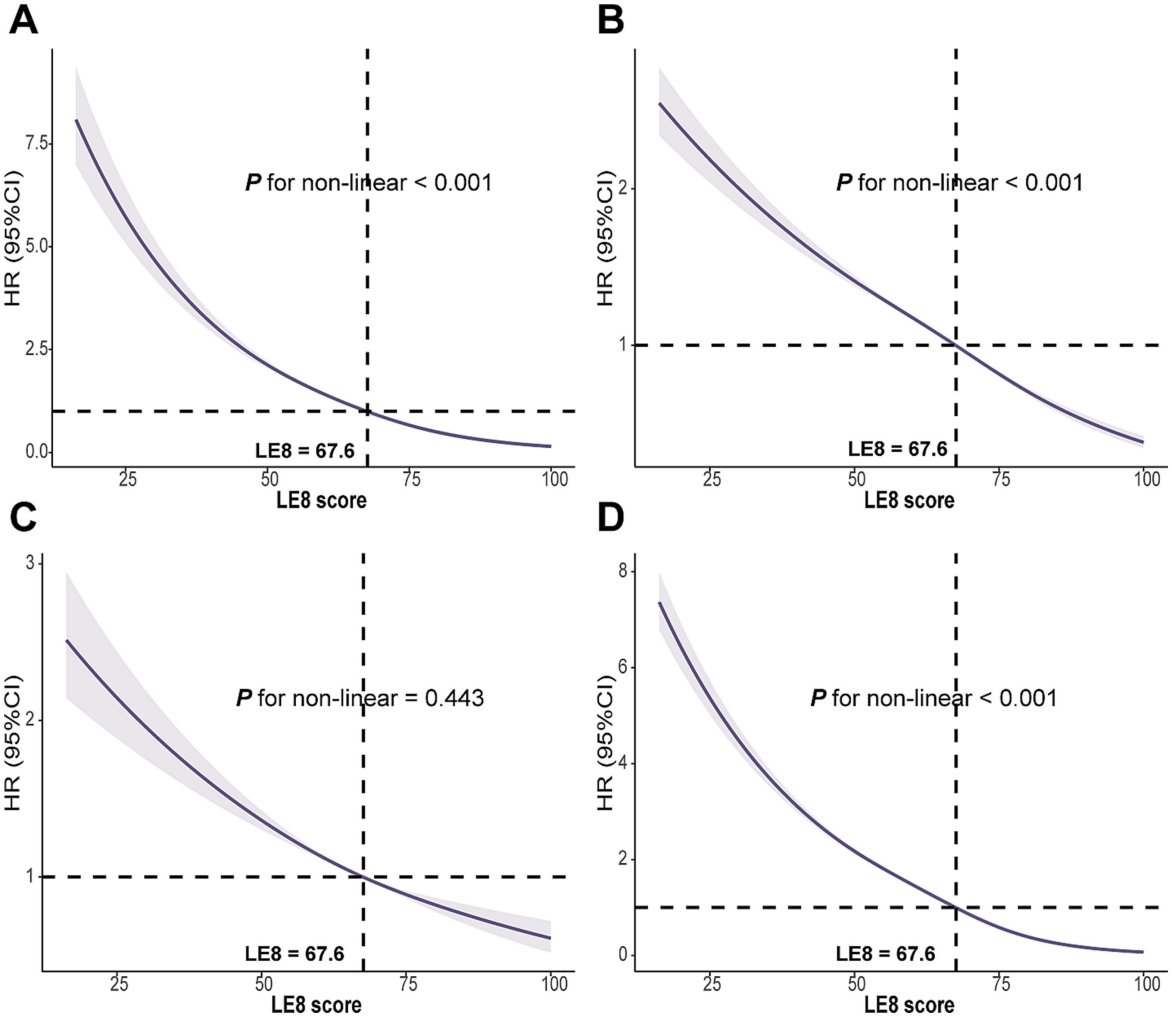
Associations between LE8 score with **(A)** CMM, **(B)** CAD, **(C)** Stroke, and **(D)** T2DM were evaluated by RCS. LE8, Life’s Essential 8; CMM, cardiometabolic multimorbidity; CAD, coronary artery disease; T2DM, type 2 diabetes mellitus; RCS, Restricted cubic spline; HR, hazard ratio; CI, confidence interval. Models were fully adjusted for age, sex, race, Townsend deprivation index, education levels, annual household income, family history of CVD, history use of lipid lowering drugs, antihypertensives, alcohol intake frequency, TyG index and CRP levels.

RCS analyses revealed a non-linear association between the LE8 score and the risks of CMM, CAD and T2DM (all *P* for non-linearity < 0.01). In contrast, a linear relationship was observed between LE8 score and stroke risk (*P* for non-linearity = 0.443). The estimated inflexion point was approximately 67.6 for the LE8 score, beyond which the reduction in CMM risk tended to plateau (Figure 2). Additionally, non-linear associations were also observed between TyG, CRP and CMM (*P* for non-linearity < 0.001; Supplementary Figure S3).

### Combination of LE8 score, TyG or CRP and the risk of CMM

**Figure 3:**
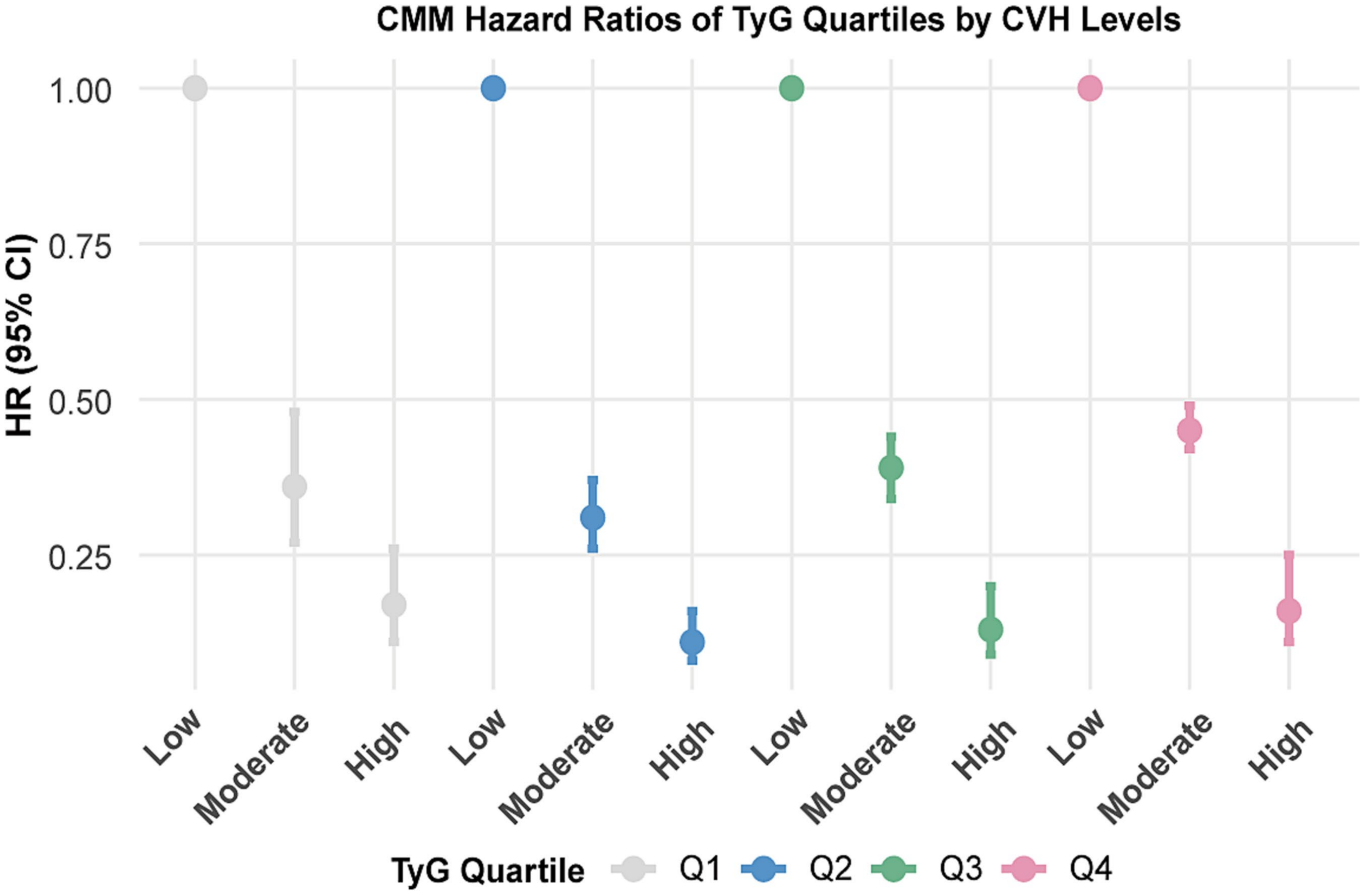
Associations between CVH groups with the risk of CMM by TyG quartiles. CVH, Cardiovascular health; CMM, cardiometabolic multimorbidity; TyG, triglyceride-glucose. Models were further adjusted for age, sex, race, Townsend deprivation index, education levels, annual household income, family history of heart diseases, history use of lipid lowering drugs, antihypertensives, alcohol intake frequency and CRP.

Table 3 summarised the combined effects of CVH groups, TyG and CRP on CMM risk. Participants with higher LE8 levels and lower TyG or CRP exposure exhibited a reduced risk of CMM (Table 3; Supplementary Figure S4). Stratified analyses across TyG and CRP quartiles indicated that the inverse association between LE8 score and CMM risk remained consistent across different levels of TyG (Figure 3) and CRP (Supplementary Figure S5).

**Table 3:** Combined effects of CVH, TyG index, CRP, and the risk of CMM.

Types	Q4	Q3	Q2	Q1
TyG index^a^				
CVH	HR (95% CI)	HR (95% CI)	HR (95% CI)	HR (95% CI)
Low	Reference	0.7 (0.62–0.78)	0.67 (0.57–0.77)	0.49 (0.38–0.62)
Moderate	0.42 (0.39–0.45)	0.25 (0.23–0.27)	0.2 (0.18–0.22)	0.15 (0.13–0.17)
High	0.19 (0.13–0.29)	0.08 (0.05–0.12)	0.07 (0.05–0.1)	0.06 (0.05–0.09)
CRP^b^				
CVH	HR (95% CI)	HR (95% CI)	HR (95% CI)	HR (95% CI)
Low	Reference	0.7 (0.62–0.78)	0.67 (0.57–0.77)	0.49 (0.38–0.62)
Moderate	0.42 (0.39–0.45)	0.25 (0.23–0.27)	0.2 (0.18–0.22)	0.15 (0.13–0.17)
High	0.19 (0.13–0.29)	0.08 (0.05–0.12)	0.07 (0.05–0.1)	0.06 (0.05–0.09)

CVH, Cardiovascular health; TyG, triglyceride-glucose; CRP, C-reactive protein; CMM, cardiometabolic multimorbidity; HR, hazard ratio; CI, confidence interval. Models were further adjusted for age, sex, race, Townsend deprivation index, education levels, annual household income, family history of heart diseases, history use of lipid lowering drugs, antihypertensives, alcohol intake frequency. ^a^Further adjusted for CRP. ^b^Further adjusted for TyG index.

### LE8 score and time to CMM onset

**Figure 4:**
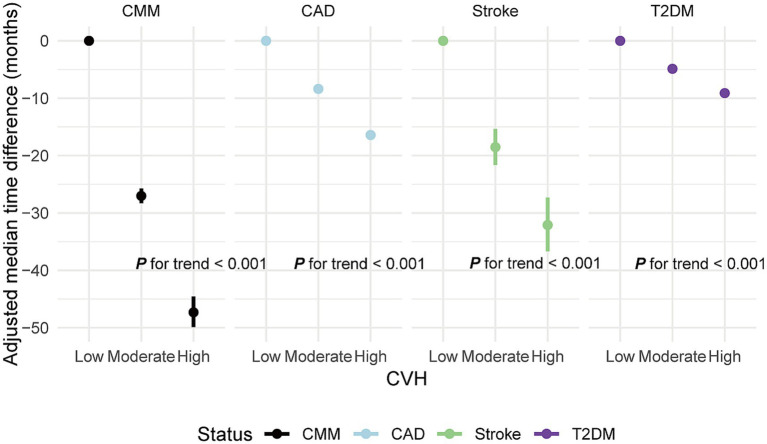
Adjusted median time difference for new-onset CMM and specific CMDs in different CVH groups. CVH, Cardiovascular health; CMM, cardiometabolic multimorbidity; CMDs, cardiometabolic diseases; CAD, coronary artery disease; T2DM, type 2 diabetes mellitus; Median difference: median occurrence time in reference group (CVH low)—median occurrence time in comparison group. Negative values indicate a delay in the onset of events, while positive values indicate an earlier onset. Models were fully adjusted for age, sex, race, Townsend deprivation index, education levels, annual household income, family history of heart diseases, history use of lipid lowering drugs, antihypertensives and alcohol intake frequency. Low CVH: CVH score < 50 points. Moderate CVH: CVH score 50–80 points. High CVH: CVH score ≥ 80 points.

AFT models indicated that higher CVH groups significantly delayed CMM onset compared to the low LE8 score group. Specifically, the time to CMM onset was delayed by 27.03 months in the moderate CVH group and by 47.32 months in the high CVH group, relative to the low group. Corresponding time ratios were 0.69 and 0.47, indicating a 31 and 53% delay in median onset time, respectively. This delay pattern was similarly observed in CAD, stroke, and T2DM (all *p* < 0.01; Figure 4; Supplementary Table S7).

### Subgroup and sensitivity analyses

**Figure 5:**
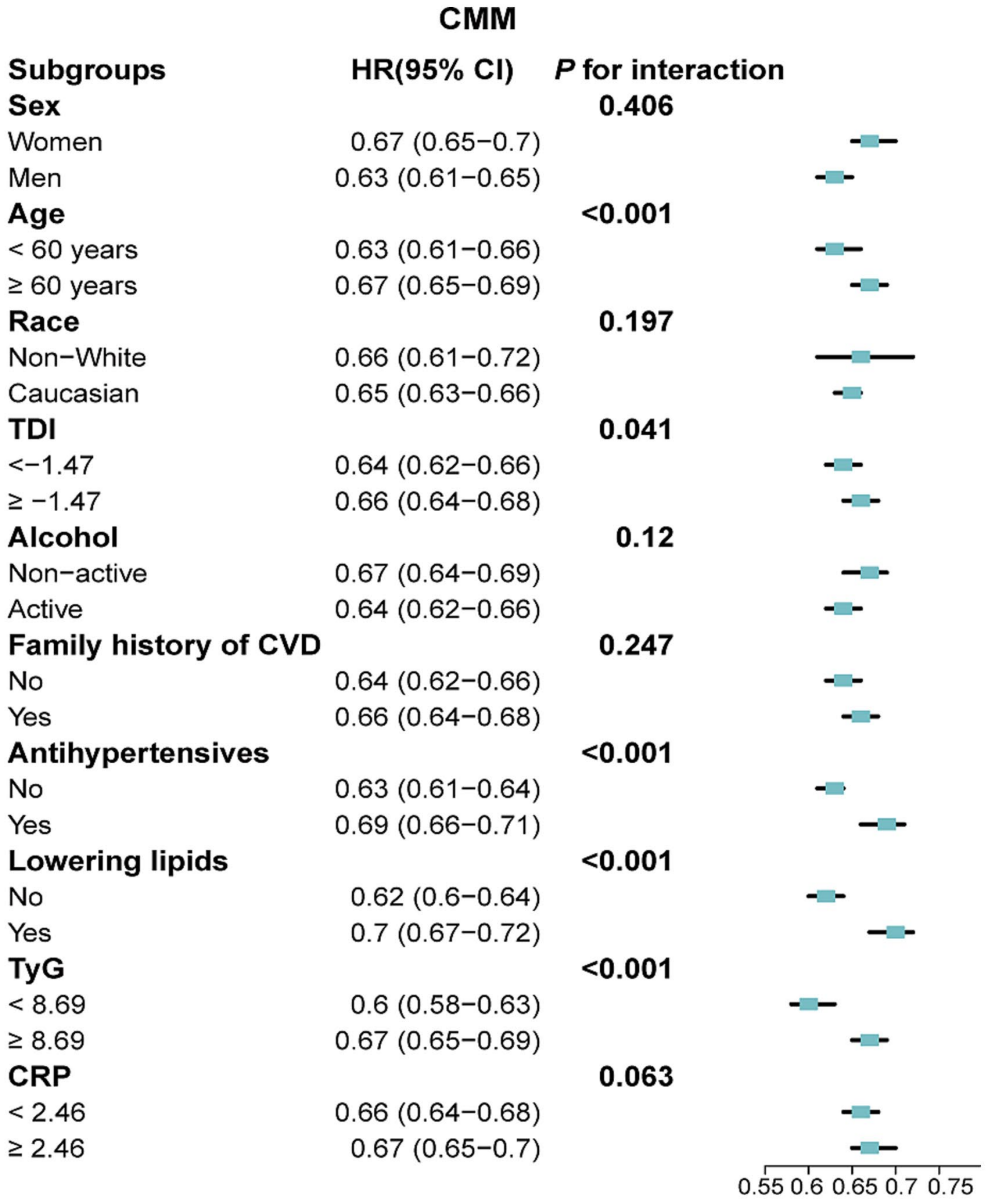
Association between a 10-point increase in LE8 score and the risk of CMM across various subgroups. LE8, Life’s Essential 8; CMM, cardiometabolic multimorbidity; TyG, triglyceride-glucose; CRP, C-reactive protein; TDI, Townsend deprivation index; HR, hazard ratio; CI, confidence interval. Models were fully adjusted for age, sex, race, TDI, education levels, annual household income, family history of CVD, history use of lipid lowering drugs, antihypertensives, alcohol intake frequency, TyG index and CRP levels.

In subgroup analyses, a 10-point increase in LE8 score was consistently linked with a reduced risk of CMM, CAD, stroke and T2DM across various demographic and clinical subgroups (Figure 5; Supplementary Figures S6–S8). A significant interaction effect was noted between each 10-point increase in LE8 score and CMM risk, with variation by age, TDI, antihypertensive use, lipid-lowering drug use, and TyG index (*P* for interaction < 0.05; Figure 5). Comparable patterns were found for CMM, CAD, stroke and T2DM when CVH was analysed across low, moderate and high groups (Supplementary Tables S8–S11). Given the older age profile of participants, we explicitly tested the potential impact of competing risks using Fine-Gray sub-distribution hazard models. The inverse association between higher LE8 scores and CMM risk remained robust (HR: 0.18; 95% CI: 0.14–0.22 for high vs. low CVH), and similar patterns were observed for CAD, stroke, and T2DM. These findings were consistent with those from the primary Cox regression models, suggesting that competing risks had minimal influence on our findings (Supplementary Table S12). Besides, the results remained robust across other sensitivity analyses (Supplementary Tables S13–S20).

### Explorative analyses

ROC curve analysis revealed that the CVH score had an AUC of 0.746 (0.74–0.752), indicating moderate predictive ability for distinguishing between individuals with and without CMM (Supplementary Figure S9). Further explorations using mediation analysis demonstrated that TyG and CRP significantly mediated the association between CVH scores and CMM risk, with mediation proportions of 18.77 and 2.86%, respectively (Supplementary Tables S21, S22; Supplementary Figure S10). Additionally, the mediation effects were slightly attenuated but remained directionally consistent. Tests for exposure–mediator interactions showed no substantial violations of the assumption (P for CVH × TyG = 0.18; CVH × CRP = 0.052) (Supplementary Tables S21, S22). Moreover, no significant interaction was found between the two mediators in the outcome model (TyG × CRP, *p* = 0.960), suggesting they act independently in mediating the effect of CVH on CMM (Supplementary Table S23).

## Discussion

This large prospective cohort study was the first to assess the independent and combined effects of LE8, TyG index and CRP levels on CMM. Our findings indicated that higher LE8 scores, coupled with lower TyG and CRP levels, were statistically associated with a lower risk of CMM. We also identified a non-linear relationship between LE8 scores, TyG, CRP and CMM risk, and found that higher CVH scores were linked to a later onset of CMM and other CMDs. The protective effect of the LE8 score on CMM persisted across multiple subgroups. Notably, the association between higher LE8 scores and lower CMM risk was partially mediated through reductions in TyG and CRP levels. Additionally, the inverse association between LE8 scores and CMM remained robust in competing risks analyses using Fine-Gray models, as well as in multiple sensitivity analyses, further strengthening our main findings.

Previous studies have indicated that combined lifestyle factors can predict CMD risk (10, 31). Additionally, the AHA’s “Life’s Simple 7” (LS7) has been linked to reduced risk of various CMDs, such as atrial fibrillation (AF), myocardial infarction and stroke (32, 33). As an expanded CVH metric, the LE8 algorithm has further strengthened overall CVH assessment and CVD risk prediction compared to LS7 (34).

The LE8 metric, as an easily accessible measure, has broad applications in CVD risk assessment. Li et al. demonstrated that each SD increase in LE8 significantly reduced CAD risk by 25% and stroke risk by 21% (35). Similarly, Zhang J et al. found that for each point increase in LE8, the risk of AF decreased by 2% (36). Additionally, another study indicated that a decrease in LE8 score significantly increased the mortality risk in patients with CVD (37). These findings, in line with our results, indicate that higher LE8 scores are associated with a lower likelihood of developing CMM. Although achieving an ideal LE8 score is challenging, our study showed that every 10-point increase in LE8 corresponds to a 35% reduction in CMM risk. Encouraging gradual lifestyle improvements aligns with AHA guidelines for CMD prevention (11).

Subgroup analyses indicated that LE8 had a significant impact on individuals under the age of 60, those not using lipid-lowering and antihypertensive medications, and participants with lower levels of TDI, TyG and CRP. This may be due to the relatively low baseline risk in these subgroups. Conversely, the weaker correlation observed in older adults, individuals on medication and participants with higher levels of TyG and CRP levels may stem from other risk factors and survival effects, which reduce the protective impact of higher CVH levels on CMM (9, 38, 39). These findings suggest that increasing LE8 levels may yield the most significant benefits for individuals under 60, who are not using lipid-lowering and/or antihypertensive medications and have lower levels of TDI and TyG. Importantly, sensitivity analyses showed the inverse association between LE8 scores and CMM risk remained robust in Fine-Gray models that accounted for death as a competing risk, which is particularly relevant given the substantial impact of mortality on CMM incidence in older adults. Besides, the consistency of our primary findings was further supported by other sensitivity analyses.

Additionally, we observed that elevated TyG and CRP levels were associated with an increased risk of CMM. For instance, Zhang Z et al. reported a 54% higher CMM risk per one-point increase in TyG (40). Similarly, Xiao D et al. observed a 45% increased CMM risk with per one SD increase in TyG index (16). Another study linked higher CRP levels with an increased risk of CMM (41). Although these studies were conducted in Asian populations, their findings align with our observations in this European cohort.

The potential pathways underlying the observed association between LE8 scores and CMM risk remain to be clarified. In our mediation analysis, the TyG index accounted for 18.8% of the association between LE8 and CMM risk, while CRP explained a smaller proportion of 2.9%. Although statistically significant, this modest mediation proportion through CRP suggests that inflammation may only partially explain the observed association, highlighting the likely involvement of other pathways such as IR. The more prominent mediation role of TyG is biologically plausible, as it serves as a surrogate marker of IR which is a central mechanism in CMM development involving endothelial dysfunction, dyslipidemia, and impaired glucose regulation (15, 42). IR has been strongly associated with diabetes, coronary artery disease, and stroke, which together define CMM (16, 40). In contrast, CRP likely captures downstream systemic inflammation secondary to metabolic disturbances, which may explain its comparatively limited mediation contribution (41, 43, 44). Given that both mediators were assessed only at baseline and evaluated independently, the observed mediation effects should be interpreted as exploratory. Moreover, the lack of repeated measurements for LE8, TyG, and CRP limits our ability to account for intra-individual changes in health behaviours and metabolic profiles over time, which may lead to exposure misclassification and attenuated associations. This limitation is particularly relevant to mediation analysis because the absence of temporally resolved data prevents confirmation of the chronological sequence among exposure, mediator, and outcome, which is essential for valid causal interpretation.

IR impairs insulin receptor activation and signal transduction, reducing cellular insulin sensitivity, leading to metabolic dysfunctions and increasing susceptibility to diseases such as T2DM (45). Furthermore, elevated insulin levels in IR stimulate lipid peroxidation and reactive oxygen species (ROS) production, activating inflammatory pathways and worsening endothelial dysfunction, which fosters CMD onset (46). Inflammatory factors like CRP and interleukin-6 (IL-6) further exacerbate insulin signalling, induce white blood cell proliferation and migration and enhance inflammatory responses (47). Chronic inflammation contributes to endothelial dysfunction, impairing the body’s antithrombotic and anti-atherosclerotic defences, promoting plaque formation and rupture and heightening the risk of atherosclerosis and CMDs (43, 48). The components of the LE8 score, such as healthy BMI, controlled lipid and glucose levels, physical activity and reduced tobacco use, are known to mitigate IR and inflammation, thus reducing CMD event risk (49–52).

A key strength of this study is its large prospective design, providing strong evidence for the relationship between CVH and CMM risk. Additionally, we identified a significant synergistic effect among LE8, TyG and CRP, advancing our understanding of LE8’s preventive role in CMM.

However, this study has several limitations. First, participant data were derived from the UK Biobank, potentially introducing selection bias, as individuals with existing CAMDs might have been less inclined to participate, thus resulting in an underestimation of CMM incidence. Second, all covariates were measured only at baseline and were not updated during follow-up. Although this is common in UK Biobank-based studies due to the limited availability of repeated measures, this approach might fail to capture changes in medication use, metabolic status, and lifestyle factors over time, potentially causing covariate misclassification and residual confounding. Third, the dietary component of the LE8 score was constructed using a binary scoring method based on nine dietary components, consistent with previous studies (53–55). While this method facilitates comparability and interpretability, it oversimplifies dietary behaviours by equally weighting each component. Future research could employ alternative approaches, such as factor analysis, to better reflect complex dietary patterns. Fourth, our definition of CMM included only CAD, T2DM, and stroke, excluding important conditions such as heart failure and chronic kidney disease, which might lead to an underestimation of the true cardiometabolic burden. Fifth, as with many large volunteer-based cohorts, the UK Biobank may be subject to healthy volunteer bias, given that participants are generally healthier, better educated, and more socioeconomically advantaged than the broader UK population. This may limit the generalizability of absolute risk estimates.

Additionally, LE8, TyG, and CRP were measured only at baseline, and potential longitudinal changes in these indicators were not captured. Such limitations could result in exposure misclassification and underestimation of time-varying effects. Given that cardiometabolic profiles and lifestyle behaviours dynamically evolve over time, our results should be interpreted cautiously. Furthermore, due to the observational nature of this study, causality between LE8, TyG, CRP, and CMM risk cannot be established. Lastly, the UK Biobank cohort predominantly comprises participants of European ancestry, limiting the generalizability of our findings to ethnically diverse populations. Future studies should validate these associations in more diverse cohorts.

## Conclusion

This study demonstrated that higher LE8 scores, along with lower levels of the IR index (TyG) and CRP, are significantly associated with a reduced risk of CMM. Additionally, both TyG and CRP were found to mediate the relationship between LE8 scores and CMM risk. These findings suggest that LE8 scores and biomarkers such as TyG and CRP may serve as useful indicators for stratifying CMM risk in the general population.

## Data Availability

Publicly available datasets were analysed in this study. This data can be found at: this study was conducted using the UK Biobank Resource under Application Number 107335. Data are available upon application to the UK Biobank at: https://www.ukbiobank.ac.uk.

## References

[1] Canoy D Tran J Zottoli M Ramakrishnan R Hassaine A Rao S. Association between cardiometabolic disease multimorbidity and all-cause mortality in 2 million women and men registered in UK general practices. BMC Med. (2021) 19:258. doi: 10.1186/s12916-021-02126-x, PMID: 34706724 PMC8555122

[2] Zhang D Tang X Shen P Si Y Liu X Xu Z. Multimorbidity of cardiometabolic diseases: prevalence and risk for mortality from one million Chinese adults in a longitudinal cohort study. BMJ Open. (2019) 9:e024476. doi: 10.1136/bmjopen-2018-024476, PMID: 30833320 PMC6443196

[3] Cheng X Ma T Ouyang F Zhang G Bai Y. Trends in the prevalence of Cardiometabolic multimorbidity in the United States, 1999-2018. Int J Environ Res Public Health. (2022) 19:726. doi: 10.3390/ijerph19084726, PMID: 35457593 PMC9027860

[4] Di Angelantonio E Kaptoge S Wormser D Willeit P Butterworth AS Bansal N. Association of cardiometabolic multimorbidity with mortality. JAMA. (2015) 314:52–60. doi: 10.1001/jama.2015.7008, PMID: 26151266 PMC4664176

[5] Fishbook BN Brinton CD Siever J Klassen TD Sakakibara BM. Cardiometabolic multimorbidity and activity limitation: a cross-sectional study of adults using the Canadian longitudinal study on aging data. Fam Pract. (2022) 39:455–63. doi: 10.1093/fampra/cmab129, PMID: 34644392

[6] Wikström K Linna M Reissell E Laatikainen T. Multimorbidity transitions and the associated healthcare cost among the Finnish adult population during a two-year follow-up. J Multimorbidity Comorbidity. (2023) 13:26335565231202325. doi: 10.1177/26335565231202325, PMID: 37711666 PMC10498690

[7] Singh-Manoux A Fayosse A Sabia S Tabak A Shipley M Dugravot A. Clinical, socioeconomic, and behavioural factors at age 50 years and risk of cardiometabolic multimorbidity and mortality: a cohort study. PLoS Med. (2018) 15:e1002571. doi: 10.1371/journal.pmed.1002571, PMID: 29782486 PMC5962054

[8] Sakakibara BM Obembe AO Eng JJ. The prevalence of cardiometabolic multimorbidity and its association with physical activity, diet, and stress in Canada: evidence from a population-based cross-sectional study. BMC Public Health. (2019) 19:1361. doi: 10.1186/s12889-019-7682-4, PMID: 31651286 PMC6814029

[9] Han Y Hu Y Yu C Guo Y Pei P Yang L. Lifestyle, cardiometabolic disease, and multimorbidity in a prospective Chinese study. Eur Heart J. (2021) 42:3374–84. doi: 10.1093/eurheartj/ehab413, PMID: 34333624 PMC8423468

[10] Freisling H Viallon V Lennon H Bagnardi V Ricci C Butterworth AS. Lifestyle factors and risk of multimorbidity of cancer and cardiometabolic diseases: a multinational cohort study. BMC Med. (2020) 18:5. doi: 10.1186/s12916-019-1474-7, PMID: 31918762 PMC6953215

[11] Lloyd-Jones DM Allen NB Anderson CA Black T Brewer LC Foraker RE. Life's essential 8: updating and enhancing the American Heart Association's construct of cardiovascular health: a presidential advisory from the American Heart Association. Circulation. (2022) 146:e18–43. doi: 10.1161/CIR.0000000000001078, PMID: 35766027 PMC10503546

[12] Rempakos A Prescott B Mitchell GF Vasan RS Xanthakis V. Association of Life's essential 8 with cardiovascular disease and mortality: the Framingham heart study. J Am Heart Assoc. (2023) 12:e030764. doi: 10.1161/JAHA.123.030764, PMID: 38014669 PMC10727315

[13] Shen R Guo X Zou T Ma L. Associations of cardiovascular health assessed by life's essential 8 with diabetic retinopathy and mortality in type 2 diabetes. Prim Care Diabetes. (2023) 17:420–8. doi: 10.1016/j.pcd.2023.08.001, PMID: 37573230

[14] Wu S Wu Z Yu D Chen S Wang A Wang A. Life's essential 8 and risk of stroke: a prospective community-based study. Stroke. (2023) 54:2369–79. doi: 10.1161/STROKEAHA.123.042525, PMID: 37466001

[15] Abdul-Ghani MA Jayyousi A DeFronzo RA Asaad N Al-Suwaidi J. Insulin resistance the link between T2DM and CVD: basic mechanisms and clinical implications. Curr Vasc Pharmacol. (2019) 17:153–63. doi: 10.2174/1570161115666171010115119, PMID: 29032755

[16] Xiao D Sun H Chen L Li X Huo H Zhou G. Assessment of six surrogate insulin resistance indexes for predicting cardiometabolic multimorbidity incidence in Chinese middle-aged and older populations: insights from the China health and retirement longitudinal study. Diabetes Metab Res Rev. (2024) 40:e3764. doi: 10.1002/dmrr.3764, PMID: 38287717

[17] Sudlow C Gallacher J Allen N Beral V Burton P Danesh J. UK biobank: an open access resource for identifying the causes of a wide range of complex diseases of middle and old age. PLoS Med. (2015) 12:e1001779. doi: 10.1371/journal.pmed.1001779, PMID: 25826379 PMC4380465

[18] Petermann-Rocha F Ho FK Foster H Boopor J Parra-Soto S Gray SR. Nonlinear associations between cumulative dietary risk factors and cardiovascular diseases, Cancer, and all-cause mortality: a prospective cohort study from UK biobank. Mayo Clin Proc. (2021) 96:2418–31. doi: 10.1016/j.mayocp.2021.01.036, PMID: 34366141

[19] Nayak R VK R Satheesh P Shenoy MT Kalra S. Triglyceride glucose (TyG) index: a surrogate biomarker of insulin resistance. JPMA J Pak Med Assoc. (2022) 72:986–8. doi: 10.47391/JPMA.22-63 35713073

[20] Lee JJ Wedow R Okbay A Kong E Maghzian O Zacher M. Gene discovery and polygenic prediction from a genome-wide association study of educational attainment in 1.1 million individuals. Nat Genet. (2018) 50:1112–21. doi: 10.1038/s41588-018-0147-3, PMID: 30038396 PMC6393768

[21] Air UK. Modelled background pollution data. London: Department for Environment, Food and Rural Affairs (2022).

[22] Pang M Platt RW Schuster T Abrahamowicz M. Spline-based accelerated failure time model. Stat Med. (2021) 40:481–97. doi: 10.1002/sim.8786, PMID: 33105513

[23] Xu S Wen S Yang Y He J Yang H Qu Y. Association between body composition patterns, cardiovascular disease, and risk of neurodegenerative disease in the UK biobank. Neurology. (2024) 103:e209659. doi: 10.1212/WNL.0000000000209659, PMID: 39047204 PMC11314951

[24] Qureshi D Collister J Allen NE Kuźma E Littlejohns T. Association between metabolic syndrome and risk of incident dementia in UK biobank. Alzheimers Dement. (2024) 20:447–58. doi: 10.1002/alz.13439, PMID: 37675869 PMC10916994

[25] Rask-Andersen M Ivansson E Höglund J Ek WE Karlsson T Johansson Å. Adiposity and sex-specific cancer risk. Cancer Cell. (2023) 41:1186–1197.e4. doi: 10.1016/j.ccell.2023.05.010, PMID: 37311415

[26] Rauscher FG Elze T Francke M Martinez-Perez ME Li Y Wirkner K. Glucose tolerance and insulin resistance/sensitivity associate with retinal layer characteristics: the LIFE-adult-study. Diabetologia. (2024) 67:928–39. doi: 10.1007/s00125-024-06093-9, PMID: 38431705 PMC10954961

[27] Uysal E Tammo O Soylemez E Incebıyık M Filiz D Alci M. Significance of measuring anthropometric and atherogenic indices in patients with polycystic ovary syndrome. BMC Endocr Disord. (2024) 24:160. doi: 10.1186/s12902-024-01701-6, PMID: 39198818 PMC11351255

[28] Russo E Viazzi F Pontremoli R Angeli F Barbagallo CM Berardino B. Predictive value of TG/HDL-C and GFR-adjusted uric acid levels on cardiovascular mortality: the URRAH study. Lipids Health Dis. (2025) 24:21. doi: 10.1186/s12944-025-02440-w, PMID: 39856749 PMC11760098

[29] Mellen PB Gao SK Vitolins MZ Goff DC. Deteriorating dietary habits among adults with hypertension: DASH dietary accordance, NHANES 1988-1994 and 1999-2004. Arch Intern Med. (2008) 168:308–14. doi: 10.1001/archinternmed.2007.119 18268173

[30] Imai K Keele L Tingley D. A general approach to causal mediation analysis. Psychol Methods. (2010) 15:309–34. doi: 10.1037/a0020761, PMID: 20954780

[31] Da H Yang R Liang J Wang J Yang W Dunk MM. Association of a low-inflammatory diet with survival among adults: the role of cardiometabolic diseases and lifestyle. Clin Nutr. (2024) 43:943–50. doi: 10.1016/j.clnu.2024.02.022, PMID: 38422952

[32] Rawal S Johnson BR Young HN Gaye B Sattler EL. Association of Life's simple 7 and ideal cardiovascular health in American Indians/Alaska natives. Open heart. (2023) 10:2222. doi: 10.1136/openhrt-2022-002222, PMID: 37024244 PMC10083851

[33] Te Hoonte F Spronk M Sun Q Wu K Fan S Wang Z. Ideal cardiovascular health and cardiovascular-related events: a systematic review and meta-analysis. Eur J Prev Cardiol. (2024) 31:966–85. doi: 10.1093/eurjpc/zwad405, PMID: 38149986

[34] Ning H Perak AM Siddique J Wilkins JT Lloyd-Jones DM Allen NB. Association between life's essential 8 cardiovascular health metrics with cardiovascular events in the cardiovascular disease lifetime risk pooling project. Circ Cardiovasc Qual Outcomes. (2024) 17:e010568. doi: 10.1161/CIRCOUTCOMES.123.010568, PMID: 38639077 PMC11209842

[35] Li X Ma H Wang X Feng H Qi L. Life's essential 8, genetic susceptibility, and incident cardiovascular disease: a prospective study. Arterioscler Thromb Vasc Biol. (2023) 43:1324–33. doi: 10.1161/ATVBAHA.123.319290, PMID: 37199161 PMC10330462

[36] Zhang J Chen G Xia H Wang X Wang C Cai M. Associations of life's essential 8 and fine particulate matter pollution with the incidence of atrial fibrillation. J Hazard Mater. (2023) 459:132114. doi: 10.1016/j.jhazmat.2023.132114 37494795

[37] Kong X Wang W. Association between life's essential 8 and all-cause or cardiovascular-specific mortality in patients with rheumatoid arthritis. Clin Exp Rheumatol. (2024) 42:1459–66. doi: 10.55563/clinexprheumatol/ppsp71, PMID: 38607691

[38] Curb JD Abbott RD Rodriguez BL Sakkinen P Popper JS Yano K. C-reactive protein and the future risk of thromboembolic stroke in healthy men. Circulation. (2003) 107:2016–20. doi: 10.1161/01.CIR.0000065228.20100.F7, PMID: 12681999

[39] Otieno P Asiki G Wekesah F Wilunda C Sanya RE Wami W. Multimorbidity of cardiometabolic diseases: a cross-sectional study of patterns, clusters and associated risk factors in sub-Saharan Africa. BMJ Open. (2023) 13:e064275. doi: 10.1136/bmjopen-2022-064275, PMID: 36759029 PMC9923299

[40] Zhang Z Zhao L Lu Y Meng X Zhou X. Relationship of triglyceride-glucose index with cardiometabolic multi-morbidity in China: evidence from a national survey. Diabetol Metab Syndr. (2023) 15:226. doi: 10.1186/s13098-023-01205-8, PMID: 37926824 PMC10626797

[41] Xu Q Tian X Xia X Zhang Y Zheng M Wang A. Estimated glucose disposal rate, high sensitivity C-reactive protein and cardiometabolic multimorbidity in middle-aged and older Chinese adults: a nationwide prospective cohort study. Diabetes Res Clin Pract. (2024) 217:111894. doi: 10.1016/j.diabres.2024.111894, PMID: 39414087

[42] Handzlik MK Gengatharan JM Frizzi KE McGregor GH Martino C Rahman G. Insulin-regulated serine and lipid metabolism drive peripheral neuropathy. Nature. (2023) 614:118–24. doi: 10.1038/s41586-022-05637-6, PMID: 36697822 PMC9891999

[43] Sinisalo J Paronen J Mattila KJ Syrjälä M Alfthan G Palosuo T. Relation of inflammation to vascular function in patients with coronary heart disease. Atherosclerosis. (2000) 149:403–11. doi: 10.1016/s0021-9150(99)00333-0, PMID: 10729391

[44] Wan B Wang S Hu S Han W Qiu S Zhu L. The comprehensive effects of high-sensitivity C-reactive protein and triglyceride glucose index on cardiometabolic multimorbidity. Front Endocrinol. (2025) 16:1511319. doi: 10.3389/fendo.2025.1511319, PMID: 40235659 PMC11996647

[45] Zhang J Jin J Liu J He Y Zhang P Ye W. A study of the correlation of insulin resistance and leptin with inflammatory factors and vascular endothelial injury in T2DM patients with CHD. Exp Ther Med. (2018) 16:265–9. doi: 10.3892/etm.2018.6170, PMID: 29896248 PMC5995089

[46] Chen W Wang X Chen J You C Ma L Zhang W. Household air pollution, adherence to a healthy lifestyle, and risk of cardiometabolic multimorbidity: results from the China health and retirement longitudinal study. Sci Total Environ. (2023) 855:158896. doi: 10.1016/j.scitotenv.2022.158896, PMID: 36150596

[47] Gainsford T Willson TA Metcalf D Handman E McFarlane C Ng A. Leptin can induce proliferation, differentiation, and functional activation of hemopoietic cells. Proc Natl Acad Sci USA. (1996) 93:14564–8. doi: 10.1073/pnas.93.25.14564, PMID: 8962092 PMC26173

[48] Di Bonito P Pacifico L Chiesa C Invitti C Del Miraglia Giudice E Baroni MG. White blood cell count may identify abnormal cardiometabolic phenotype and preclinical organ damage in overweight/obese children. Nutr Metab Cardiovasc Dis. (2016) 26:502–9. doi: 10.1016/j.numecd.2016.01.013 27048715

[49] Coniglio RI Meroño T Montiel H Malaspina MM Salgueiro AM Otero JC. HOMA-IR and non-HDL-C as predictors of high cholesteryl ester transfer protein activity in patients at risk for type 2 diabetes. Clin Biochem. (2012) 45:566–70. doi: 10.1016/j.clinbiochem.2012.02.005, PMID: 22366373

[50] Kitahara CM Trabert B Katki HA Chaturvedi AK Kemp TJ Pinto LA. Body mass index, physical activity, and serum markers of inflammation, immunity, and insulin resistance. Cancer Epidemiol Biomark Prevent. (2014) 23:2840–9. doi: 10.1158/1055-9965.EPI-14-0699-T PMC4257882 25249326

[51] Mukharjee S Bank S Maiti S. Chronic tobacco exposure by smoking develops insulin resistance. Endocr Metab Immune Disord Drug Targets. (2020) 20:869–77. doi: 10.2174/1871530320666200217123901, PMID: 32065107

[52] Han H Wang Y Li T Feng C Kaliszewski C Su Y. Sleep duration and risks of incident cardiovascular disease and mortality among people with type 2 diabetes. Diabetes Care. (2023) 46:101–10. doi: 10.2337/dc22-1127, PMID: 36383480

[53] Cheng W Du Z Lu B. Chronic low-grade inflammation associated with higher risk and earlier onset of cardiometabolic multimorbidity in middle-aged and older adults: a population-based cohort study. Sci Rep. (2024) 14:22635. doi: 10.1038/s41598-024-72988-7, PMID: 39349699 PMC11442589

[54] Yu W Wang X Du Z Cheng W. Association of triglyceride-glucose index and its combination with obesity indicators in predicting the risk of aortic aneurysm and dissection. Front Nutr. (2024) 11:1454880. doi: 10.3389/fnut.2024.1454880, PMID: 39507901 PMC11537997

[55] Yang C Cheng W Plum PS Lordick F Köppe J Gockel I. Life's essential 8 and specific cancer risk and mortality in men and women: a population-based cohort analysis of 332,417 United Kingdom participants. BMC Cancer. (2025) 25:632. doi: 10.1186/s12885-025-14048-5, PMID: 40200269 PMC11980174

