# Supplementary material for: Exploring the synergistic effects of Life’s Essential 8, insulin resistance, and CRP on cardiometabolic multimorbidity risk
Source: Front Nutr. 2025 Jul 16;12:1598659. doi: 10.3389/fnut.2025.1598659 (PMC12307207; doi:10.3389/fnut.2025.1598659)
Supplement: Supplementary file 1 [file Table_1.docx]

**Exploring the synergistic effects of Life’s Essential 8, insulin resistance, and CRP on cardiometabolic multimorbidity risk**

**Jiang Liu, Chuang Yang, Wenke Cheng, Daidi Li**

[Table S1. Assessment Methods for Each Individual CVH Metric. 3](#_Toc201718637)

[Table S2. UK Biobank self‐report highest qualification mapped to international standard for classification of education codes. 5](#_Toc201718638)

[Table S3. Information on missing covariates among 304,568 UK Biobank participants. 6](#_Toc201718639)

[Table S4. Baseline characteristics comparison between included and excluded participants. 7](#_Toc201718640)

[Table S5. Baseline characteristics comparison between included cohort and general population. 8](#_Toc201718641)

[Table S6. The association between TyG index, CRP and the risk of CMM. 9](#_Toc201718642)

[Table S7. Adjusted Absolute Time Differences and Time Ratios for CMM, CAD, Stroke, and T2DM by CVH Level. 10](#_Toc201718643)

[Table S8. Association between CVH groups and the risk of CMM stratified by various subgroups. 11](#_Toc201718644)

[Table S9. Association between CVH groups and the risk of CAD stratified by various subgroups. 12](#_Toc201718645)

[Table S10. Association between CVH groups and the risk of Stroke stratified by various subgroups. 13](#_Toc201718646)

[Table S11. Association between CVH groups and the risk of T2DM stratified by various subgroups. 14](#_Toc201718647)

[Table S12. Fine-Gray proportional hazard regression models for the association between LE8 score and the risk of CMM and specific CMDs. 15](#_Toc201718648)

[Table S13. The association between LE8 score and the risk of CMM and specific CMDs after excluding the participants with follow up within 2 years. 16](#_Toc201718649)

[Table S14. The association between the LE8 score and the risk of CMM and specific CMDs was analyzed after excluding participants with missing covariate values at baseline. 17](#_Toc201718650)

[Table S15. Association between LE8 score and risk of CMM and specific CMDs after excluding participants with a history of antihypertensive and lipid-lowering medication use. 18](#_Toc201718651)

[Table S16. Association between LE8 score and the risk of CMM and specific CMDs without adjustment for TyG and CRP levels. 19](#_Toc201718652)

[Table S17. Association between LE8 score and the risk of CMM and specific CMDs with further adjustment for PM_2.5_, PM_2.5-10_, PM_10_, NO_2_ and NO_x_. 20](#_Toc201718653)

[Table S18. Association between LE8 score categories and the risk of CMM and specific CMDs after multiple imputations of other four datasets. 21](#_Toc201718654)

[Table S19. Associations of alternative insulin resistance indices with the risk of CMM. 23](#_Toc201718655)

[Table S20. The association between LE8 score and the risk of CMM and specific CMDs. 24](#_Toc201718656)

[Table S21. Mediation analysis results of CVH on CMM risk through TyG. 25](#_Toc201718657)

[Table S22. Mediation analysis results of CVH on CMM risk through CRP. 26](#_Toc201718658)

[Table S23. Association of the interaction between TyG and CRP with risk of CMM. 27](#_Toc201718659)

[Figure S1. Schoenfeld residual plots for assessing proportional hazards assumption. 28](#_Toc201718660)

[Figure S2. Kaplan–Meier survival curves illustrating the association between CVH groups and the risk of CMM (A), CAD (B), Stroke (C) and T2DM (D). 29](#_Toc201718661)

[Figure S3. Associations between TyG (A), CRP (B) with CMM were evaluated by RCS. 30](#_Toc201718662)

[Figure S4. Combined effects of CVH, TyG or CRP and the risk of CMM. 31](#_Toc201718663)

[Figure S5. Associations between CVH groups with the risk of CMM by CRP quartiles. 32](#_Toc201718664)

[Figure S6. Association between a 10-point increase in LE8 score and the risk of CAD across various subgroups. 33](#_Toc201718665)

[Figure S7. Association between a 10-point increase in LE8 score and the risk of Stroke across various subgroups. 34](#_Toc201718666)

[Figure S8. Association between a 10-point increase in LE8 score and the risk of T2DM across various subgroups. 35](#_Toc201718667)

[Figure S9. The discriminatory power of LE8 score for the development of CMM. 36](#_Toc201718668)

[Figure S10. Mediation effect to TyG (A) or CRP (B) on the relationship between LE8 score and the risk of CMM. 37](#_Toc201718669)

[Appendix 38](#_Toc201718670)

| Table S1. Assessment Methods for Each Individual CVH Metric. | | | |
| --- | --- | --- | --- |
| CVH metric ^2^ | Points | Diet score | Metrics: Diet score |
| Diet score | 100 | 0-2 | The self-completed touch-screen question-naire (completed at baseline) was used to collect the frequency of consumption of food items during the previous year. 9 food items were dichotomized into meeting and  not meeting recommendations using cut-offs.  1-point was assigned to participants for each unhealthy category met^1^. |
|  | 80 | 3-4 |  |
|  | 50 | 5 |  |
|  | 25 | 6 |  |
|  | 0 | 7-9 |  |
| Physical activity score | Points | Physical activity | Metrics: minutes of moderate- intensity activity per week |
|  | 100 | ≥150 minutes | Participants reported their weekly minutes of moderate or vigorous physical activity. One minute of vigorous activity is deemed equivalent to two minutes of moderate activity. |
|  | 90 | 120-149 minutes |  |
|  | 80 | 90-119 minutes |  |
|  | 60 | 60-89 minutes |  |
|  | 40 | 30-59 minutes |  |
|  | 20 | 1-29 minutes |  |
|  | 0 | 0 minutes |  |
| Smoking score | Points | Smoking | Metrics: Tobacco/nicotine  /Secondhand smoke exposure |
|  | 100 | Never smoker | Participants self-reported their tobacco use, encompassing current smoking status and smoking history, as well as exposure to secondhand smoke. Participants living with an active indoor smoker at home had 20 points deducted from their score, unless their score was zero.  Participants who indicated they 'smoked on most or all days in the past.' were classified as “Former smoker, quit <1 years”.  Participants who indicated they 'smoked occasionally in the past' were classified as “Former smoker, quit 1-5 years”.  Participants who reported having 'just tried once or twice in the past' were classified as “Former smoker, quit ≥ 5 years”. |
|  | 75 | Former smoker, quit ≥ 5years |  |
|  | 50 | Former smoker, quit 1-5years |  |
|  | 25 | Former smoker, quit < 1 years |  |
|  | 0 | Current smoker |  |
| Sleep health score | Points | Sleep duration | Metrics: Sleep hours |
|  | 100 | 7-9 hours | Participants reported their average nightly sleep duration. |
|  | 90 | 9-10 hours |  |
|  | 70 | 6-7 hours |  |
|  | 40 | 5-6 or ≥10 hours |  |
|  | 20 | 4–5 hours |  |
|  | 0 | < 4 hours |  |
| Body mass index score | Points | Body mass index | Metrics: Body mass index |
|  | 100 | <25 kg/m^2^ | Height was determined using the Seca 202 height measuring instrument. Weight was precisely measured to the nearest 0.1 kg using the Tanita BC-418 MA body composition analyzer. Body mass index (BMI) was calculated as weight (in kg) divided by the square of height (in meters). |
|  | 70 | 25.0-30.0 kg/m^2^ |  |
|  | 30 | 30.0-35 kg/m^2^ |  |
|  | 15 | 35.0-40 kg/m^2^ |  |
|  | 0 | ≥ 40 kg/m^2^ |  |
| Blood lipid score | Point | Non-HDL cholesterol | Metrics: Non-HDL cholesterol |
|  | 100 | < 130 mg/dL | Non-HDL cholesterol was derived by subtracting HDL cholesterol from total cholesterol. Serum cholesterol levels were determined enzymatically.  If treated with medication, deduct 20 points. |
|  | 60 | 130-159 mg/dL |  |
|  | 40 | 160-189 mg/dL |  |
|  | 20 | 190-219 mg/dL |  |
|  | 0 | 220 mg/dL |  |
| Blood glucose score | Points | HbA1c | Metrics: HbA1c |
|  | 100 | No diabetes and HbA1c<5.7% | HbA1c levels were determined using high-performance liquid chromatography techniques. |
|  | 60 | No diabetes and HbA1c 5.7–6.4% |  |
|  | 50 | No diabetes and HbA1c >6.4% |  |
|  | 40 | Diabetes with HbA1c<7.0% |  |
|  | 30 | Diabetes with HbA1c 7.0-8.0% |  |
|  | 20 | Diabetes with HbA1c 8.0-9.0% |  |
|  | 10 | Diabetes with HbA1c 9.0-10.0% |  |
|  | 0 | Diabetes with HbA1c≥10.0 |  |
| Blood pressure score | Points | Blood pressure | Metrics: Systolic and diastolic BPs |
|  | 100 | <120 / <80 mmHg | The mean of all available blood pressure readings was utilized to determine both systolic and diastolic blood pressures.  Subtract 20 points from the score (unless the score is 0) if under treatment. |
|  | 75 | 120-130 / < 80 mmHg |  |
|  | 50 | 130-140 or 80-90 mmHg |  |
|  | 25 | 140-160 or 90-100 mmHg |  |
|  | 0 | ≥ 160 or ≥ 100 mmHg |  |

| Table S2. UK Biobank self‐report highest qualification mapped to international standard for classification of education codes. | |
| --- | --- |
| **Qualification (As reported in UK Biobank)** | **ISCED** |
| College or University degree | 4 |
| NVQ or HND or HNC or equivalent | 4 |
| Other prof. qual. eg: nursing, teaching | 3 |
| A levels/AS levels or equivalent | 2 |
| O levels/GCSEs or equivalent | 1 |
| CSEs or equivalent | 1 |
| None of the above | 0 |

| Table S3. Information on missing covariates among 304,568 UK Biobank participants. |
| --- |

| **Covariates** | **Number of missing** | **Missing rates (%)** |
| --- | --- | --- |
| Race and ethnicity | 879 | 0 |
| Townsend Deprivation Index | 377 | 0 |
| Education levels | 2,243 | 1 |
| Annual household income before tax | 36,972 | 12 |
| Antihypertensives | 15 | 0 |
| Lowering lipids drugs | 15 | 0 |
| Alcohol intake frequency | 132 | 0 |
| TyG | 487 | 0 |
| CRP | 638 | 16 |
| PM_2.5_ | 25,440 | 8 |
| PM_2.5-10_ | 25,440 | 8 |
| PM_10_ | 25,440 | 8 |
| NO_2_ | 4,129 | 1 |
| NO_x_ | 4,129 | 1 |

TyG: triglyceride-glucose; CRP:C-reactive protein;

| Table S4. Baseline characteristics comparison between included and excluded participants. |
| --- |

| **Characteristics** | **Included** | **Excluded** | ***P*** |
| --- | --- | --- | --- |
| N | 304,568 | 197,787 |  |
| Age, years | 55.75 ± 8.09 | 57.74 ± 7.95 | <0.001 |
| Male | 164,689 (54.1%) | 89,185 (45.1%) | <0.001 |
| White | 290,477 (95.4%) | 184,679 (93.4%) | <0.001 |
| Townsend deprivation index | -1.47 ± 2.99 | -1.03 ± 3.23 | <0.001 |
| Education levels |  |  | <0.001 |
| CSEs or equivalent | 43,384 (14.2%) | 44,083 (22.3%) |  |
| A levels/AS levels or equivalent | 82,625 (27.1%) | 52,060 (26.3%) |  |
| Other professional qualification | 36,156 (11.9%) | 20,004 (10.1%) |  |
| College or University degree | 15,367 (5.1%) | 10,899 (5.5%) |  |
| None of the above | 127,036 (41.7%) | 70,741 (35.8%) |  |
| Annual household income before tax, £ |  |  | <0.001 |
| <18,000 | 62,169 (20.4%) | 60,108 (30.4%) |  |
| 18,000-30,999 | 75,734 (24.9%) | 53,443 (27.0%) |  |
| 31 000-51,999 | 82,282 (27.0%) | 45,681 (23.1%) |  |
| 52,000-100,000 | 66,486 (21.8%) | 30,971 (15.7%) |  |
| >100,000 | 17,897 (5.9%) | 7,584 (3.8%) |  |
| History of heart diseases family | 118,789 (39.0%) | 81,076 (41.0%) | <0.001 |
| Lipid-lowering drugs | 35,769 (11.7%) | 51,629 (26.1%) | <0.001 |
| Antihypertensives | 49,353 (16.2%) | 55,284 (28.0%) | <0.001 |
| Alcohol intake frequency |  |  | <0.001 |
| Never | 21,183 (7.0%) | 19,604 (9.9%) |  |
| Special occasions only | 31,841 (10.5%) | 26,341 (13.3%) |  |
| One to three times a month | 33,640 (11.1%) | 22,372 (11.3%) |  |
| Once or twice a week | 79,649 (26.2%) | 49,989 (25.3%) |  |
| Three or four times a week | 73,936 (24.3%) | 41,782 (21.1%) |  |
| Daily or almost daily | 64,319 (21.1%) | 37,699 (19.1%) |  |
| TyG index | 8.69 ± 0.56 | 8.76 ± 0.57 | <0.001 |
| CRP (mmol/l) | 2.46 ± 4.12 | 2.83 ± 4.71 | <0.001 |

CSE: Certificate of Secondary Education; AS levels: Advanced Subsidiary Level Education; TyG: triglyceride-glucose; CRP:C-reactive protein;

| Table S5. Baseline characteristics comparison between included cohort and general population. |
| --- |

| **Characteristics** | **Included cohort** | **General population** | **SMD** |
| --- | --- | --- | --- |
| N | 304,568 | 502,355 |  |
| Age, years | 55.75 ± 8.09 | 56.53 ± 8.09 | 0.249 |
| Male | 164,689 (54.1%) | 229,064 (45.6%) | 0.017 |
| White | 290,477 (95.4%) | 475,164 (94.6%) | 0.087 |
| Townsend deprivation index | -1.47 ± 2.99 | -1.29 ± 3.10 | 0.14 |
| Education levels |  |  | 0.167 |
| CSEs or equivalent | 43,384 (14.2%) | 87,548 (17.4%) |  |
| A levels/AS levels or equivalent | 82,625 (27.1%) | 134,715 (26.8%) |  |
| Other professional qualification | 36,156 (11.9%) | 56,116 (11.2%) |  |
| College or University degree | 15,367 (5.1%) | 26,257 (5.2%) |  |
| None of the above | 127,036 (41.7%) | 197,719 (39.4%) |  |
| Annual household income before tax, £ |  |  | 0.262 |
| <18,000 | 62,169 (20.4%) | 123,760 (24.6%) |  |
| 18,000-30,999 | 75,734 (24.9%) | 129,275 (25.7%) |  |
| 31 000-51,999 | 82,282 (27.0%) | 127,378 (25.4%) |  |
| 52,000-100,000 | 66,486 (21.8%) | 96,732 (19.3%) |  |
| >100,000 | 17,897 (5.9%) | 25,210 (5.0%) |  |
| History of heart diseases family | 118,789 (39.0%) | 199,865 (39.8%) | 0.041 |
| Lipid-lowering drugs | 35,769 (11.7%) | 87,399 (17.4%) | 0.373 |
| Antihypertensives | 49,353 (16.2%) | 104,638 (20.8%) | 0.286 |
| Alcohol intake frequency |  |  | 0.144 |
| Never | 21,183 (7.0%) | 40,783 (8.1%) |  |
| Special occasions only | 31,841 (10.5%) | 58,188 (11.6%) |  |
| One to three times a month | 33,640 (11.1%) | 56,012 (11.1%) |  |
| Once or twice a week | 79,649 (26.2%) | 129,635 (25.8%) |  |
| Three or four times a week | 73,936 (24.3%) | 115,713 (23.0%) |  |
| Daily or almost daily | 64,319 (21.1%) | 102,024 (20.3%) |  |
| TyG index | 8.69 ± 0.56 | 8.72 ± 0.56 | 0.129 |
| CRP (mmol/l) | 2.46 ± 4.12 | 2.61 ± 4.37 | 0.082 |

SMD: standardized mean difference; CSE: Certificate of Secondary Education; AS levels: Advanced Subsidiary Level Education; TyG: triglyceride-glucose; CRP:C-reactive protein;

| Table S6. The association between TyG index, CRP and the risk of CMM. |
| --- |

| **Type** | **Model 1** | | **Model 2** | |
| --- | --- | --- | --- | --- |
|  | **HR (95%CI)** | ***P*** | **HR (95%CI)** | ***P*** |
| **TyG^a^** |  |  |  |  |
| Q1 | Reference |  | Reference |  |
| Q2 | 1.59 (1.42-1.78) | < 0.001 | 1.23 (1.1-1.38) | < 0.001 |
| Q3 | 2.25 (2.02-2.50) | < 0.001 | 1.41 (1.26-1.57) | < 0.001 |
| Q4 | 4.51 (4.08-4.99) | < 0.001 | 2.08 (1.87-2.31) | < 0.001 |
| *P* for trend | < 0.001 |  | < 0.001 |  |
| Per SD increase | 1.84 (1.79-1.88) | < 0.001 | 1.39 (1.35-1.43) | < 0.001 |
| **CRP^b^** |  |  |  |  |
| Q1 | Reference |  | Reference |  |
| Q2 | 1.38 (1.25-1.53) | < 0.001 | 1 (0.90-1.11) | 0.982 |
| Q3 | 1.90 (1.73-2.09) | < 0.001 | 1.11 (1.01-1.22) | 0.036 |
| Q4 | 3.55 (3.25-3.88) | < 0.001 | 1.58 (1.44-1.73) | < 0.001 |
| *P* for trend | < 0.001 |  | < 0.001 |  |
| Per SD increase | 1.18 (1.17-1.20) | < 0.001 | 1.12 (1.1-1.14) | < 0.001 |

TyG: triglyceride-glucose; CRP:C-reactive protein; CMM: cardiometabolic multimorbidity; HR: hazard ratio; CI: confidence interval; Model 1 adjusted age, sex, and race. Model 2 was further adjusted for Townsend deprivation index, education levels, annual household income, family history of heart diseases, history use of lipid lowering drugs, antihypertensives, alcohol intake frequency and LE8 score. ^a^: further adjusted for CRP in model 2. ^b^: further adjusted for TyG index in model 2.

| Table S7. Adjusted Absolute Time Differences and Time Ratios for CMM, CAD, Stroke, and T2DM by CVH Level. | | |
| --- | --- | --- |
| **Categories** | **Absolute time difference (months)** | **Time ratio** |
| CVH | **CMM** | |
| Low |  |  |
| Moderate | -27.03 (-28.31 to -25.72) | 0.69 (0.68 to 0.71) |
| High | -47.32 (-49.9 to -44.57) | 0.47 (0.44 to 0.50) |
| CVH | **CAD** | |
| Low | Ref | |
| Moderate | -8.38 (-8.91 to -7.84) | 0.84 (0.83 to 0.85) |
| High | -16.42 (-17.23 to -15.58) | 0.68 (0.67 to 0.70) |
| CVH | **Stroke** | |
| Low | Ref | |
| Moderate | -18.53 (-21.64 to -15.35) | 0.65 (0.64 to 0.66) |
| High | -32.1 (-36.7 to -27.32) | 0.34 (0.33 to 0.36) |
| CVH | **T2DM** | |
| Low | Ref | |
| Moderate | -4.88 (-5.0 to -4.76) | 0.88 (0.86 to 0.90) |
| High | -9.12 (-9.33 to -8.9) | 0.80 (0.77 to 0.83) |

CVH: Cardiovascular health; CMM: cardiometabolic multimorbidity; CAD: coronary artery disease; T2DM: type 2 diabetes mellitus; CI: confidence interval. Absolute time differences (in months) were calculated based on adjusted AFT (Weibull) models using CVH low group as reference. Time ratios were derived as exp(β) from the same models, representing the ratio of median survival time in the Low group compared to the Moderate and High CVH groups. The difference between 1 and the time ratio reflects the percentage of delay in disease occurrence. Models were fully adjusted for age, sex, race, Townsend deprivation index, education levels, annual household income, family history of heart diseases, history use of lipid lowering drugs, antihypertensives and alcohol intake frequency. Low CVH: LE8 score < 50 points. Moderate CVH: LE8 score 50-80 points. High CVH: LE8 score ≥ 80 points.

| Table S8. Association between CVH groups and the risk of CMM stratified by various subgroups. |
| --- |

| **Subgroups** | **Low** | **Moderate** | **High** |  |  |
| --- | --- | --- | --- | --- | --- |
|  | **HR (95% CI)** | **HR (95% CI)** | **HR (95% CI)** | ***P* for trend** | ***P* for interaction** |
| Sex |  |  |  |  | 0.392 |
| Women | Reference | 0.46 (0.42-0.52) | 0.18 (0.14-0.24) | <0.001 |  |
| Men | Reference | 0.42 (0.39-0.45) | 0.17 (0.13-0.22) | <0.001 |  |
| Age |  |  |  |  | <0.001 |
| < 60 years | Reference | 0.42 (0.38-0.46) | 0.15 (0.11-0.2) | <0.001 |  |
| ≥ 60 years | Reference | 0.46 (0.43-0.5) | 0.19 (0.15-0.24) | <0.001 |  |
| Race |  |  |  |  | 0.219 |
| Non-White | Reference | 0.49 (0.39-0.62) | 0.21 (0.12-0.37) | <0.001 |  |
| Caucasian | Reference | 0.44 (0.41-0.47) | 0.17 (0.14-0.2) | <0.001 |  |
| TDI |  |  |  |  | 0.177 |
| <-1.47 | Reference | 0.42 (0.38-0.46) | 0.17 (0.14-0.22) | <0.001 |  |
| ≥ -1.47 | Reference | 0.46 (0.43-0.5) | 0.16 (0.12-0.22) | <0.001 |  |
| Alcohol intake frequency | |  |  |  | 0.205 |
| Non-active | Reference | 0.46 (0.41-0.5) | 0.2 (0.16-0.26) | <0.001 |  |
| Active | Reference | 0.43 (0.4-0.47) | 0.15 (0.11-0.19) | <0.001 |  |
| Family history of CVD | |  |  |  | 0.078 |
| No | Reference | 0.44 (0.4-0.48) | 0.14 (0.11-0.18) | <0.001 |  |
| Yes | Reference | 0.45 (0.41-0.49) | 0.2 (0.16-0.26) | <0.001 |  |
| Antihypertensives | |  |  |  | <0.001 |
| No | Reference | 0.4 (0.37-0.44) | 0.18 (0.14-0.22) | <0.001 |  |
| Yes | Reference | 0.49 (0.44-0.53) | 0.15 (0.1-0.23) | <0.001 |  |
| Lowering lipids | |  |  |  | <0.001 |
| No | Reference | 0.39 (0.36-0.43) | 0.18 (0.14-0.22) | <0.001 |  |
| Yes | Reference | 0.53 (0.48-0.58) | 0.15 (0.1-0.22) | <0.001 |  |
| TyG |  |  |  |  | <0.001 |
| < 8.69 | Reference | 0.33 (0.29-0.38) | 0.14 (0.11-0.18) | <0.001 |  |
| ≥ 8.69 | Reference | 0.47 (0.44-0.51) | 0.17 (0.13-0.23) | <0.001 |  |
| CRP |  |  |  |  | 0.87 |
| < 2.46 | Reference | 0.48 (0.44-0.53) | 0.2 (0.16-0.25) | <0.001 |  |
| ≥ 2.46 | Reference | 0.46 (0.43-0.5) | 0.19 (0.13-0.27) | <0.001 |  |

CVH: Cardiovascular health; CMM: cardiometabolic multimorbidity; HR: hazard ratio; CI: confidence interval. Models were fully adjusted for age, sex, race, Townsend deprivation index, education levels, annual household income, family history of heart diseases, history use of lipid lowering drugs, antihypertensives, alcohol intake frequency, TyG index and CRP levels. Low CVH: LE8 score < 50 points. Moderate CVH: LE8 score 50-80 points. High CVH: LE8 score ≥ 80 points.

| Table S9. Association between CVH groups and the risk of CAD stratified by various subgroups. |
| --- |

| **Subgroups** | **Low** | **Moderate** | **High** |  |  |
| --- | --- | --- | --- | --- | --- |
|  | **HR (95% CI)** | **HR (95% CI)** | **HR (95% CI)** | ***P* for trend** | ***P* for interaction** |
| Sex |  |  |  |  | <0.001 |
| Women | Reference | 0.65 (0.61-0.69) | 0.42 (0.38-0.46) | <0.001 |  |
| Men | Reference | 0.7 (0.67-0.73) | 0.44 (0.4-0.48) | <0.001 |  |
| Age |  |  |  |  | <0.001 |
| < 60 years | Reference | 0.63 (0.6-0.67) | 0.36 (0.33-0.39) | <0.001 |  |
| ≥ 60 years | Reference | 0.74 (0.71-0.78) | 0.53 (0.49-0.58) | <0.001 |  |
| Race |  |  |  |  | 0.848 |
| Non-White | Reference | 0.68 (0.57-0.8) | 0.46 (0.34-0.61) | <0.001 |  |
| Caucasian | Reference | 0.69 (0.66-0.72) | 0.43 (0.41-0.46) | <0.001 |  |
| TDI |  |  |  |  | 0.444 |
| <-1.47 | Reference | 0.7 (0.66-0.73) | 0.46 (0.42-0.5) | <0.001 |  |
| ≥ -1.47 | Reference | 0.69 (0.65-0.72) | 0.41 (0.37-0.45) | <0.001 |  |
| Alcohol intake frequency | |  |  |  | 0.001 |
| Non-active | Reference | 0.63 (0.59-0.67) | 0.4 (0.36-0.44) | <0.001 |  |
| Active | Reference | 0.72 (0.69-0.76) | 0.46 (0.43-0.5) | <0.001 |  |
| Family history of CVD | |  |  |  | 0.1 |
| No | Reference | 0.68 (0.65-0.71) | 0.4 (0.37-0.44) | <0.001 |  |
| Yes | Reference | 0.7 (0.67-0.74) | 0.48 (0.44-0.52) | <0.001 |  |
| Antihypertensives | |  |  |  | <0.001 |
| No | Reference | 0.65 (0.63-0.68) | 0.4 (0.38-0.44) | <0.001 |  |
| Yes | Reference | 0.74 (0.7-0.79) | 0.61 (0.54-0.71) | <0.001 |  |
| Lowering lipids | |  |  |  | <0.001 |
| No | Reference | 0.67 (64-0.7) | 0.41 (0.38-0.44) | <0.001 |  |
| Yes | Reference | 0.74 (0.69-0.79) | 0.6 (0.52-0.68) | <0.001 |  |
| TyG |  |  |  |  | <0.001 |
| < 8.69 | Reference | 0.61 (0.57-0.66) | 0.39 (0.36-0.43) | <0.001 |  |
| ≥ 8.69 | Reference | 0.71 (0.68-0.74) | 0.48 (0.43-0.53) | <0.001 |  |
| CRP |  |  |  |  | 0.128 |
| < 2.46 | Reference | 0.72 (0.69-0.76) | 0.49 (0.45-0.53) | <0.001 |  |
| ≥ 2.46 | Reference | 0.71 (0.67-0.75) | 0.43 (0.38-0.49) | <0.001 |  |

CVH: Cardiovascular health; CMM: cardiometabolic multimorbidity; HR: hazard ratio; CI: confidence interval. Models were fully adjusted for age, sex, race, Townsend deprivation index, education levels, annual household income, family history of heart diseases, history use of lipid lowering drugs, antihypertensives, alcohol intake frequency, TyG index and CRP levels. Low CVH: LE8 score < 50 points. Moderate CVH: LE8 score 50-80 points. High CVH: LE8 score ≥ 80 points.

| Table S10. Association between CVH groups and the risk of Stroke stratified by various subgroups. |
| --- |

| **Subgroups** | **Low** | **Moderate** | **High** |  | ***P* for interaction** |
| --- | --- | --- | --- | --- | --- |
|  | **HR (95% CI)** | **HR (95% CI)** | **HR (95% CI)** | ***P* for trend** |  |
| Sex |  |  |  |  | 0.138 |
| Women | Reference | 0.76 (0.67-0.85) | 0.55 (0.47-0.65) | <0.001 |  |
| Men | Reference | 0.69 (0.63-0.75) | 0.56 (0.48-0.65) | <0.001 |  |
| Age |  |  |  |  | 0.003 |
| < 60 years | Reference | 0.65 (0.58-0.73) | 0.5 (0.42-0.59) | <0.001 |  |
| ≥ 60 years | Reference | 0.76 (0.69-0.82) | 0.58 (0.51-0.67) | <0.001 |  |
| Race |  |  |  |  | 0.873 |
| Non-White | Reference | 0.82 (0.56-1.19) | 0.67 (0.38-1.19) | <0.001 |  |
| Caucasian | Reference | 0.72 (0.67-0.77) | 0.54 (0.49-0.61) | <0.001 |  |
| TDI |  |  |  |  | 0.225 |
| <-1.47 | Reference | 0.75 (0.68-0.82) | 0.58 (0.5-0.66) | <0.001 |  |
| ≥ -1.47 | Reference | 0.7 (0.63-0.77) | 0.52 (0.44-0.61) | <0.001 |  |
| Alcohol intake frequency | |  |  |  | 0.938 |
| Non-active | Reference | 0.71 (0.63-0.8) | 0.53 (0.44-0.64) | <0.001 |  |
| Active | Reference | 0.73 (0.67-0.79) | 0.56 (0.49-0.63) | <0.001 |  |
| Family history of CVD | |  |  |  | 0.261 |
| No | Reference | 0.73 (0.67-0.8) | 0.6 (0.52-0.69) | <0.001 |  |
| Yes | Reference | 0.71 (0.63-0.79) | 0.48 (0.41-0.57) | <0.001 |  |
| Antihypertensives | |  |  |  | 0.032 |
| No | Reference | 0.7 (0.64-0.76) | 0.53 (0.47-0.6) | <0.001 |  |
| Yes | Reference | 0.74 (0.66-0.83) | 0.64 (0.51-0.82) | <0.001 |  |
| Lowering lipids | |  |  |  | 0.416 |
| No | Reference | 0.75 (0.69-0.81) | 0.58 (0.51-0.65) | <0.001 |  |
| Yes | Reference | 0.64 (0.56-0.73) | 0.44 (0.34-0.58) | <0.001 |  |
| TyG |  |  |  |  | 0.626 |
| < 8.69 | Reference | 0.71 (0.62-0.81) | 0.56 (0.47-0.66) | <0.001 |  |
| ≥ 8.69 | Reference | 0.73 (0.67-0.79) | 0.51 (0.42-0.61) | <0.001 |  |
| CRP |  |  |  |  | 0.287 |
| < 2.46 | Reference | 0.77 (0.7-0.86) | 0.6 (0.53-0.69) | <0.001 |  |
| ≥ 2.46 | Reference | 0.71 (0.65-0.78) | 0.52 (0.42-0.65) | <0.001 |  |

CVH: Cardiovascular health; CMM: cardiometabolic multimorbidity; HR: hazard ratio; CI: confidence interval. Models were fully adjusted for age, sex, race, Townsend deprivation index, education levels, annual household income, family history of heart diseases, history use of lipid lowering drugs, antihypertensives, alcohol intake frequency, TyG index and CRP levels. Low CVH: LE8 score < 50 points. Moderate CVH: LE8 score 50-80 points. High CVH: LE8 score ≥ 80 points.

| Table S11. Association between CVH groups and the risk of T2DM stratified by various subgroups. |
| --- |

| **Subgroups** | **Low** | **Moderate** | **High** |  |  |
| --- | --- | --- | --- | --- | --- |
|  | **HR (95% CI)** | **HR (95% CI)** | **HR (95% CI)** | ***P* for trend** | ***P* for interaction** |
| Sex |  |  |  |  | 0.015 |
| Women | Reference | 0.44 (0.42-0.47) | 0.13 (0.11-0.15) | <0.001 |  |
| Men | Reference | 0.44 (0.42-0.46) | 0.12 (0.1-0.15) | <0.001 |  |
| Age |  |  |  |  | 0.001 |
| < 60 years | Reference | 0.44 (0.42-0.46) | 0.12 (0.1-0.13) | <0.001 |  |
| ≥ 60 years | Reference | 0.46 (0.44-0.49) | 0.14 (0.12-0.16) | <0.001 |  |
| Race |  |  |  |  | <0.001 |
| Non-White | Reference | 0.58 (0.51-0.65) | 0.16 (0.12-0.21) | <0.001 |  |
| Caucasian | Reference | 0.44 (0.42-0.45) | 0.12 (0.11-0.14) | <0.001 |  |
| TDI |  |  |  |  | <0.001 |
| <-1.47 | Reference | 0.42 (0.4-0.44) | 0.12 (0.1-0.14) | <0.001 |  |
| ≥ -1.47 | Reference | 0.47 (0.45-0.49) | 0.13 (0.11-0.16) | <0.001 |  |
| Alcohol intake frequency | |  |  |  | <0.001 |
| Non-active | Reference | 0.49 (0.47-0.52) | 0.13 (0.11-0.15) | <0.001 |  |
| Active | Reference | 0.42 (0.4-0.44) | 0.12 (0.1-0.14) | <0.001 |  |
| Family history of CVD | |  |  |  | 0.1 |
| No | Reference | 0.43 (0.42-0.45) | 0.12 (0.1-0.14) | <0.001 |  |
| Yes | Reference | 0.46 (0.44-0.49) | 0.14 (0.12-0.16) | <0.001 |  |
| Antihypertensives | |  |  |  | <0.001 |
| No | Reference | 0.4 (0.38-0.42) | 0.12 (0.11-0.14) | <0.001 |  |
| Yes | Reference | 0.52 (0.49-0.55) | 0.13 (0.11-0.17) | <0.001 |  |
| Lowering lipids | |  |  |  | <0.001 |
| No | Reference | 0.39 (0.37-0.4) | 0.12 (0.11-0.14) | <0.001 |  |
| Yes | Reference | 0.56 (0.53-0.59) | 0.14 (0.11-0.17) | <0.001 |  |
| TyG |  |  |  |  | <0.001 |
| < 8.69 | Reference | 0.34 (0.32-0.38) | 0.1 (0.08-0.11) | <0.001 |  |
| ≥ 8.69 | Reference | 0.47 (0.45-0.48) | 0.14 (0.12-0.16) | <0.001 |  |
| CRP |  |  |  |  | 0.128 |
| < 2.46 | Reference | 0.49 (0.46-0.51) | 0.16 (0.14-0.18) | <0.001 |  |
| ≥ 2.46 | Reference | 0.47 (0.45-0.49) | 0.12 (0.1-0.15) | <0.001 |  |

CVH: Cardiovascular health; CMM: cardiometabolic multimorbidity; HR: hazard ratio; CI: confidence interval. Models were fully adjusted for age, sex, race, Townsend deprivation index, education levels, annual household income, family history of heart diseases, history use of lipid lowering drugs, antihypertensives, alcohol intake frequency, TyG index and CRP levels. Low CVH: LE8 score < 50 points. Moderate CVH: LE8 score 50-80 points. High CVH: LE8 score ≥ 80 points.

| Table S12. Fine-Gray proportional hazard regression models for the association between LE8 score and the risk of CMM and specific CMDs. |
| --- |

| **Type** | **HR (95%CI)** | ***P*** |
| --- | --- | --- |
| **CMM** |  |  |
| Low | Reference |  |
| Moderate | 0.38 (0.34-0.41) | < 0.001 |
| High | 0.18 (0.14-0.22) | < 0.001 |
| *P* for trend | <0.001 |  |
| Per 10-point increase | 0.61 (0.59-0.63) | < 0.001 |
| **CAD** |  |  |
| Low | Reference |  |
| Moderate | 0.64 (0.61-0.67) | < 0.001 |
| High | 0.38 (0.35-0.41) | < 0.001 |
| *P* for trend | <0.001 |  |
| Per 10-point increase | 0.78 (0.77-0.8) | < 0.001 |
| **Stroke** |  |  |
| Low | Reference |  |
| Moderate | 0.71 (0.65-0.78) | < 0.001 |
| High | 0.55 (0.48-0.62) | < 0.001 |
| *P* for trend | <0.001 |  |
| Per 10-point increase | 0.84 (0.82-0.86) | < 0.001 |
| **T2DM** |  |  |
| Low | Reference |  |
| Moderate | 0.36 (0.34-0.38) | < 0.001 |
| High | 0.12 (0.1-0.14) | < 0.001 |
| *P* for trend | <0.001 |  |
| Per 10-point increase | 0.58 (0.57-0.59) | < 0.001 |

CVH: Cardiovascular health; CMM: cardiometabolic multimorbidity; CMDs: cardiometabolic diseases; CAD: coronary artery disease; T2DM: type 2 diabetes mellitus; HR: hazard ratio; CI: confidence interval. Models were fully adjusted for age, sex, race, Townsend deprivation index, education levels, annual household income, family history of heart diseases, history use of lipid lowering drugs, antihypertensives, alcohol intake frequency, TyG index and CRP levels. Low CVH: LE8 score < 50 points. Moderate CVH: LE8 score 50-80 points. High CVH: LE8 score ≥ 80 points.

| Table S13. The association between LE8 score and the risk of CMM and specific CMDs after excluding the participants with follow up within 2 years. |
| --- |

| **Type** | **HR (95% CI)** | ***P*** |
| --- | --- | --- |
| **CMM** |  |  |
| Low | Reference |  |
| Moderate | 0.44 (0.41-0.47) | < 0.001 |
| High | 0.16 (0.14-0.2) | < 0.001 |
| *P* for trend | <0.001 |  |
| Per 10-point increase | 0.65 (0.63-0.66) | < 0.001 |
| **CAD** |  |  |
| Low | Reference |  |
| Moderate | 0.69 (0.67-0.72) | < 0.001 |
| High | 0.43 (0.4-0.46) | < 0.001 |
| *P* for trend | <0.001 |  |
| Per 10-point increase | 0.81 (0.8-0.82) | < 0.001 |
| **Stroke** |  |  |
| Low | Reference |  |
| Moderate | 0.73 (0.68-0.78) | < 0.001 |
| High | 0.56 (0.5-0.63) | < 0.001 |
| *P* for trend | <0.001 |  |
| Per 10-point increase | 0.85 (0.83-0.87) | < 0.001 |
| **T2DM** |  |  |
| Low | Reference |  |
| Moderate | 0.44 (0.42-0.45) | < 0.001 |
| High | 0.12 (0.11-0.14) | < 0.001 |
| *P* for trend | <0.001 |  |
| Per 10-point increase | 0.63 (0.63-0.64) | < 0.001 |

CVH: Cardiovascular health; CMM: cardiometabolic multimorbidity; CMDs: cardiometabolic diseases; CAD: coronary artery disease; T2DM: type 2 diabetes mellitus; HR: hazard ratio; CI: confidence interval. Models were fully adjusted for age, sex, race, Townsend deprivation index, education levels, annual household income, family history of heart diseases, history use of lipid lowering drugs, antihypertensives, alcohol intake frequency, TyG index and CRP levels. Low CVH: LE8 score < 50 points. Moderate CVH: LE8 score 50-80 points. High CVH: LE8 score ≥ 80 points.

| Table S14. The association between the LE8 score and the risk of CMM and specific CMDs was analyzed after excluding participants with missing covariate values at baseline. |
| --- |

| **Type** | **HR (95% CI)** | ***P*** |
| --- | --- | --- |
| **CMM** |  |  |
| Low | Reference |  |
| Moderate | 0.43 (0.4-0.46) | < 0.001 |
| High | 0.16 (0.13-0.19) | < 0.001 |
| *P* for trend | <0.001 |  |
| Per 10-point increase | 0.64 (0.63-0.66) | < 0.001 |
| **CAD** |  |  |
| Low | Reference |  |
| Moderate | 0.67 (0.65-0.7) | < 0.001 |
| High | 0.42 (0.39-0.45) | < 0.001 |
| *P* for trend | <0.001 |  |
| Per 10-point increase | 0.8 (0.79-0.81) | < 0.001 |
| **Stroke** |  |  |
| Low | Reference |  |
| Moderate | 0.7 (0.65-0.76) | < 0.001 |
| High | 0.54 (0.48-0.6) | < 0.001 |
| *P* for trend | <0.001 |  |
| Per 10-point increase | 0.84 (0.82-0.86) | < 0.001 |
| **T2DM** |  |  |
| Low | Reference |  |
| Moderate | 0.43 (0.42-0.45) | < 0.001 |
| High | 0.12 (0.1-0.13) | < 0.001 |
| *P* for trend | <0.001 |  |
| Per 10-point increase | 0.63 (0.62-0.64) | < 0.001 |

CVH: Cardiovascular health; CMM: cardiometabolic multimorbidity; CMDs: cardiometabolic diseases; CAD: coronary artery disease; T2DM: type 2 diabetes mellitus; HR: hazard ratio; CI: confidence interval. Models were fully adjusted for age, sex, race, Townsend deprivation index, education levels, annual household income, family history of heart diseases, history use of lipid lowering drugs, antihypertensives, alcohol intake frequency, TyG index and CRP levels. Low CVH: LE8 score < 50 points. Moderate CVH: LE8 score 50-80 points. High CVH: LE8 score ≥ 80 points.

| Table S15. Association between LE8 score and risk of CMM and specific CMDs after excluding participants with a history of antihypertensive and lipid-lowering medication use. |
| --- |

| **Type** | **HR (95%CI)** | ***P*** |
| --- | --- | --- |
| **CMM** |  |  |
| Low | Reference |  |
| Moderate | 0.33 (0.31-0.35) | < 0.001 |
| High | 0.09 (0.08-0.11) | < 0.001 |
| *P* for trend | <0.001 |  |
| Per 10-point increase | 0.58 (0.56-0.59) | < 0.001 |
| **CAD** |  |  |
| Low | Reference |  |
| Moderate | 0.62 (0.6-0.64) | < 0.001 |
| High | 0.36 (0.33-0.38) | < 0.001 |
| *P* for trend | <0.001 |  |
| Per 10-point increase | 0.78 (0.77-0.79) | < 0.001 |
| **Stroke** |  |  |
| Low | Reference |  |
| Moderate | 0.7 (0.66-0.75) | < 0.001 |
| High | 0.53 (0.48-0.59) | < 0.001 |
| *P* for trend | <0.001 |  |
| Per 10-point increase | 0.84 (0.83-0.86) | < 0.001 |
| **T2DM** |  |  |
| Low | Reference |  |
| Moderate | 0.31 (0.3-0.32) | < 0.001 |
| High | 0.05 (0.05-0.06) | < 0.001 |
| *P* for trend | <0.001 |  |
| Per 10-point increase | 0.54 (0.53-0.55) | < 0.001 |

CVH: Cardiovascular health; CMM: cardiometabolic multimorbidity; CMDs: cardiometabolic diseases; CAD: coronary artery disease; T2DM: type 2 diabetes mellitus; HR: hazard ratio; CI: confidence interval. Models were fully adjusted for age, sex, race, Townsend deprivation index, education levels, annual household income, family history of heart diseases, history use of lipid lowering drugs, antihypertensives, alcohol intake frequency, TyG index and CRP levels. Low CVH: LE8 score < 50 points. Moderate CVH: LE8 score 50-80 points. High CVH: LE8 score ≥ 80 points.

| Table S16. Association between LE8 score and the risk of CMM and specific CMDs without adjustment for TyG and CRP levels. |
| --- |

| **Type** | **HR (95%CI)** | ***P*** |
| --- | --- | --- |
| **CMM** |  |  |
| Low | Reference |  |
| Moderate | 0.44 (0.41-0.47) | < 0.001 |
| High | 0.17 (0.14-0.2) | < 0.001 |
| *P* for trend | <0.001 |  |
| Per 10-point increase | 0.65 (0.63-0.66) | < 0.001 |
| **CAD** |  |  |
| Low | Reference |  |
| Moderate | 0.68 (0.66-0.71) | < 0.001 |
| High | 0.43 (0.4-0.46) | < 0.001 |
| *P* for trend | <0.001 |  |
| Per 10-point increase | 0.81 (0.8-0.82) | < 0.001 |
| **Stroke** |  |  |
| Low | Reference |  |
| Moderate | 0.72 (0.67-0.77) | < 0.001 |
| High | 0.55 (0.49-0.61) | < 0.001 |
| *P* for trend | <0.001 |  |
| Per 10-point increase | 0.84 (0.83-0.86) | < 0.001 |
| **T2DM** |  |  |
| Low | Reference |  |
| Moderate | 0.44 (0.43-0.46) | < 0.001 |
| High | 0.12 (0.11-0.14) | < 0.001 |
| *P* for trend | <0.001 |  |
| Per 10-point increase | 0.63 (0.63-0.64) | < 0.001 |

CVH: Cardiovascular health; CMM: cardiometabolic multimorbidity; CMDs: cardiometabolic diseases; CAD: coronary artery disease; T2DM: type 2 diabetes mellitus; HR: hazard ratio; CI: confidence interval. Models were fully adjusted for age, sex, race, Townsend deprivation index, education levels, annual household income, family history of heart diseases, history use of lipid lowering drugs, antihypertensives and alcohol intake frequency. Low CVH: LE8 score < 50 points. Moderate CVH: LE8 score 50-80 points. High CVH: LE8 score ≥ 80 points.

| Table S17. Association between LE8 score and the risk of CMM and specific CMDs with further adjustment for PM_2.5_, PM_2.5-10_, PM_10_, NO_2_ and NO_x_. |
| --- |

| **Type** | **HR (95%CI)** | ***P*** |
| --- | --- | --- |
| **CMM** |  |  |
| Low | Reference |  |
| Moderate | 0.44 (0.42-0.47) | < 0.001 |
| High | 0.18 (0.15-0.21) | < 0.001 |
| *P* for trend | <0.001 |  |
| Per 10-point increase | 0.66 (0.64-0.67) | < 0.001 |
| **CAD** |  |  |
| Low | Reference |  |
| Moderate | 0.69 (0.67-0.72) | < 0.001 |
| High | 0.44 (0.41-0.47) | < 0.001 |
| *P* for trend | <0.001 |  |
| Per 10-point increase | 0.81 (0.8-0.82) | < 0.001 |
| **Stroke** |  |  |
| Low | Reference |  |
| Moderate | 0.74 (0.69-0.79) | < 0.001 |
| High | 0.56 (0.5-0.63) | < 0.001 |
| *P* for trend | <0.001 |  |
| Per 10-point increase | 0.86 (0.84-0.87) | < 0.001 |
| **T2DM** |  |  |
| Low | Reference |  |
| Moderate | 0.45 (0.43-0.46) | < 0.001 |
| High | 0.12 (0.11-0.14) | < 0.001 |
| *P* for trend | <0.001 |  |
| Per 10-point increase | 0.64 (0.63-0.65) | < 0.001 |

CVH: Cardiovascular health; CMM: cardiometabolic multimorbidity; CMDs: cardiometabolic diseases; CAD: coronary artery disease; T2DM: type 2 diabetes mellitus; HR: hazard ratio; CI: confidence interval. Models were fully adjusted for age, sex, race, Townsend deprivation index, education levels, annual household income, family history of heart diseases, history use of lipid lowering drugs, antihypertensives, alcohol intake frequency, TyG, CRP, PM2.5, PM2.5-10, PM10, NO2 and NOx. Low CVH: LE8 score < 50 points. Moderate CVH: LE8 score 50-80 points. High CVH: LE8 score ≥ 80 points.

| Table S18. Association between LE8 score categories and the risk of CMM and specific CMDs after multiple imputations of other four datasets. |
| --- |

| **Type** | **Imputation 1** | | **Imputation 2** | | **Imputation 3** | | **Imputation 4** | | **Pooled results** | |
| --- | --- | --- | --- | --- | --- | --- | --- | --- | --- | --- |
|  | **HR (95%CI)** | ***P*** | **HR (95%CI)** | ***P*** | **HR (95%CI)** | ***P*** | **HR (95%CI)** | ***P*** | **HR (95%CI)** | ***P*** |
| **CMM** |  |  |  |  |  |  |  |  |  |  |
| Low | Reference |  | Reference |  | Reference |  | Reference |  | Reference |  |
| Moderate | 0.44 (0.41-0.46) | < 0.001 | 0.44 (0.41-0.47) | < 0.001 | 0.44 (0.41-0.46) | < 0.001 | 0.44 (0.41-0.47) | < 0.001 | 0.44 (0.41–0.47) | < 0.001 |
| High | 0.17 (0.14-0.2) | < 0.001 | 0.17 (0.14-0.2) | < 0.001 | 0.17 (0.14-0.2) | < 0.001 | 0.17 (0.14-0.2) | < 0.001 | 0.17 (0.14–0.20) | < 0.001 |
| P for trend | <0.001 |  | <0.001 |  | <0.001 |  | <0.001 |  | <0.001 |  |
| Per 10-point increase | 0.65 (0.63-0.66) | < 0.001 | 0.65 (0.63-0.66) | < 0.001 | 0.65 (0.63-0.66) | < 0.001 | 0.65 (0.63-0.66) | < 0.001 | 0.65 (0.63–0.66) | < 0.001 |
| **CAD** |  |  |  |  |  |  |  |  |  |  |
| Low | Reference |  | Reference |  | Reference |  | Reference |  |  |  |
| Moderate | 0.68 (0.66-0.71) | < 0.001 | 0.68 (0.66-0.71) | < 0.001 | 0.68 (0.66-0.71) | < 0.001 | 0.68 (0.66-0.71) | < 0.001 | 0.68 (0.66–0.71) | < 0.001 |
| High | 0.43 (0.40-0.46) | < 0.001 | 0.43 (0.40-0.46) | < 0.001 | 0.43 (0.40-0.46) | < 0.001 | 0.43 (0.40-0.46) | < 0.001 | 0.43 (0.40–0.46) | < 0.001 |
| P for trend | <0.001 |  | <0.001 |  | <0.001 |  | <0.001 |  |  |  |
| Per 10-point increase | 0.81 (0.8-0.82) | < 0.001 | 0.81 (0.8-0.82) | < 0.001 | 0.81 (0.8-0.82) | < 0.001 | 0.81 (0.8-0.82) | < 0.001 | 0.81 (0.80–0.82) | < 0.001 |
| **Stroke** |  |  |  |  |  |  |  |  |  |  |
| Low | Reference |  | Reference |  | Reference |  | Reference |  |  |  |
| Moderate | 0.72 (0.67-0.77) | < 0.001 | 0.72 (0.67-0.77) | < 0.001 | 0.72 (0.67-0.77) | < 0.001 | 0.72 (0.67-0.77) | < 0.001 | 0.72 (0.67–0.77) | < 0.001 |
| High | 0.55 (0.49-0.61) | < 0.001 | 0.55 (0.49-0.61) | < 0.001 | 0.55 (0.49-0.61) | < 0.001 | 0.55 (0.49-0.61) | < 0.001 | 0.55 (0.49–0.61) | < 0.001 |
| P for trend | <0.001 |  | <0.001 |  | <0.001 |  | <0.001 |  |  |  |
| Per 10-point increase | 0.84 (0.83-0.86) | < 0.001 | 0.84 (0.83-0.86) | < 0.001 | 0.84 (0.83-0.86) | < 0.001 | 0.84 (0.83-0.86) | < 0.001 | 0.84 (0.83–0.86) | < 0.001 |
| **T2DM** |  |  |  |  |  |  |  |  |  |  |
| Low | Reference |  | Reference |  | Reference |  | Reference |  |  |  |
| Moderate | 0.44 (0.43-0.46) | < 0.001 | 0.44 (0.43-0.46) | < 0.001 | 0.44 (0.43-0.46) | < 0.001 | 0.44 (0.43-0.46) | < 0.001 | 0.44 (0.43–0.46) | < 0.001 |
| High | 0.12 (0.11-0.14) | < 0.001 | 0.12 (0.11-0.14) | < 0.001 | 0.12 (0.11-0.14) | < 0.001 | 0.12 (0.11-0.14) | < 0.001 | 0.12 (0.11–0.14) | < 0.001 |
| P for trend | <0.001 |  | <0.001 |  | <0.001 |  | <0.001 |  |  |  |
| Per 10-point increase | 0.64 (0.63-0.64) | < 0.001 | 0.64 (0.63-0.64) | < 0.001 | 0.63 (0.63-0.64) | < 0.001 | 0.63 (0.63-0.64) | < 0.001 | 0.63 (0.63–0.64) | < 0.001 |

CMM: cardiometabolic multimorbidity; CMDs: cardiometabolic diseases; CAD: coronary artery disease; T2DM: type 2 diabetes mellitus; HR: hazard ratio; CI: confidence interval. Models were fully adjusted for age, sex, race, Townsend deprivation index, education levels, annual household income, family history of heart diseases, history use of lipid lowering drugs, antihypertensives, alcohol intake frequency, TyG and CRP.

| **Type** | **HR (95%CI)** | ***P*** |
| --- | --- | --- |
| **TG/HDL-C** |  |  |
| Q1 | Reference |  |
| Q2 | 1.25 (1.11-1.40) | < 0.001 |
| Q3 | 1.42 (1.28-1.58) | < 0.001 |
| Q4 | 1.85 (1.66-2.06) | < 0.001 |
| *P* for trend | < 0.001 |  |
| Per SD increase | 1.15 (1.13-1.17) | < 0.001 |
| **METS-IR** |  |  |
| Q1 | Reference |  |
| Q2 | 1.09 (0.96-1.24) | 0.194 |
| Q3 | 1.42 (1.26-1.60) | < 0.001 |
| Q4 | 2.29 (2.03-2.58) | < 0.001 |
| *P* for trend | < 0.001 |  |
| Per SD increase | 1.39 (1.35-1.42) | < 0.001 |
| **eGDR** |  |  |
| Q1 | Reference |  |
| Q2 | 0.60 (0.56-0.65) | < 0.001 |
| Q3 | 0.53 (0.48-0.58) | < 0.001 |
| Q4 | 0.29 (0.24-0.34) | < 0.001 |
| *P* for trend | < 0.001 |  |
| Per SD increase | 0.57 (0.55-0.59) | < 0.001 |

| Table S19. Associations of alternative insulin resistance indices with the risk of CMM. |
| --- |

CMM: cardiometabolic multimorbidity; TG/HDL-C: triglyceride-to-high-density lipoprotein cholesterol ratio; METS-IR: metabolic score for insulin resistance; eGDR: estimated glucose disposal rate; HR: hazard ratio; CI: confidence interval. Models were fully adjusted for age, sex, race, Townsend deprivation index, education levels, annual household income, family history of heart diseases, history use of lipid lowering drugs, antihypertensives, alcohol intake frequency, LE8 score and CRP.

| Table S20. The association between LE8 score and the risk of CMM and specific CMDs. |
| --- |

| **Type** | **HR (95%CI)** | ***P*** |
| --- | --- | --- |
| **CMM** |  |  |
| Low | Reference |  |
| Moderate | 0.41 (0.36-0.46) | < 0.001 |
| High | 0.16 (0.11-0.23) | < 0.001 |
| P for trend | <0.001 |  |
| Per 10-point increase | 0.61 (0.58-0.64) | < 0.001 |
| **CAD** |  |  |
| Low | Reference |  |
| Moderate | 0.71 (0.66-0.75) | < 0.001 |
| High | 0.43 (0.38-0.48) | < 0.001 |
| P for trend | <0.001 |  |
| Per 10-point increase | 0.80 (0.79-0.82) | < 0.001 |
| **Stroke** |  |  |
| Low | Reference |  |
| Moderate | 0.69 (0.61-0.77) | < 0.001 |
| High | 0.57 (0.47-0.69) | < 0.001 |
| P for trend | <0.001 |  |
| Per 10-point increase | 0.85 (0.82-0.88) | < 0.001 |
| **T2DM** |  |  |
| Low | Reference |  |
| Moderate | 0.41 (0.38-0.43) | < 0.001 |
| High | 0.13 (0.10-0.16) | < 0.001 |
| P for trend | <0.001 |  |
| Per 10-point increase | 0.61 (0.59-0.62) | < 0.001 |

The LE8 diet score was constructed based on the Dietary Approaches to Stop Hypertension (DASH) index. CMM: cardiometabolic multimorbidity; CMDs: cardiometabolic diseases; CAD: coronary artery disease; T2DM: type 2 diabetes mellitus; HR: hazard ratio; CI: confidence interval. Models were fully adjusted for age, sex, race, Townsend deprivation index, education levels, annual household income, family history of heart diseases, history use of lipid lowering drugs, antihypertensives, alcohol intake frequency, TyG and CRP.

| Table S21. Mediation analysis results of CVH on CMM risk through TyG. | | | | |  |
| --- | --- | --- | --- | --- | --- |
| **Type** | **Estimate ^a^** | **95% CI ^a^** | **Estimate ^b^** | **95% CI ^b^** | ***P* for interaction** |
| Total effect | -0.0918 | -0.1002 to -0.0844 | -0.0722 | -0.0877 to -0.0600 | < 0.001 |
| Direct Effect (ADE, average) | -0.0746 | -0.082 to -0.068 | -0.0633 | -0.0749 to -0.0500 | < 0.001 |
| Indirect effect (ACME, average) | -0.0172 | -0.0189 to -0.0155 | -0.0089 | -0.0148 to 0.0000 | 0.18 |
| Proportion mediated (average) | 18.76% | 17.20% to 20.6% | 12.38% | 5.92% to 18.0% | 0.18 |

CVH: Cardiovascular health; CMM: cardiometabolic multimorbidity; TyG: triglyceride-glucose; CI: confidence interval. ADE: Average direct effect. ACME: Average causal mediation effect. ^a^: no interaction; ^b^: with interaction. Models were fully adjusted for age, sex, race, Townsend deprivation index, education levels, annual household income, family history of heart diseases, history use of lipid lowering drugs, antihypertensives, alcohol intake frequency, and CRP levels.

| Table S22. Mediation analysis results of CVH on CMM risk through CRP. | | | | | |
| --- | --- | --- | --- | --- | --- |
| **Type** | **Estimate ^a^** | **95% CI ^a^** | **Estimate ^b^** | **95% CI ^b^** | ***P* for interaction** |
| Total effect | -0.0889 | -0.097 to -0.0807 | -0.0862 | -0.0954 to -0.0800 | < 0.001 |
| Direct Effect (ADE, average) | -0.0864 | -0.0943 to -0.0785 | -0.0848 | -0.0933 to -0.0800 | < 0.001 |
| Indirect effect (ACME, average) | -0.0025 | -0.0029 to -0.0021 | -0.0013 | -0.0032 to 0.0000 | 0.052 |
| Proportion mediated (average) | 2.86% | 2.40% to 3.29% | 1.55% | -0.07% to 3.00% | 0.052 |

CVH: Cardiovascular health; CMM: cardiometabolic multimorbidity; CRP:C-reactive protein; CI: confidence interval. ADE: Average direct effect. ACME: Average causal mediation effect. ^a^: no interaction; ^b^: with interaction. Models were fully adjusted for age, sex, race, Townsend deprivation index, education levels, annual household income, family history of heart diseases, history use of lipid lowering drugs, antihypertensives, alcohol intake frequency, and TyG levels.

| Table S23. Association of the interaction between TyG and CRP with risk of CMM. | | | | |
| --- | --- | --- | --- | --- |
| **Variable** | **Coefficient (β)** | **SE** | **z value** | ***P-*value** |
| TyG | 0.625 | 0.003 | 20.93 | <0.001 |
| CRP | 0.029 | 0.030 | 0.88 | 0.379 |
| TyG × CRP (interaction term) | –0.00019 | 0.004 | –0.050 | 0.96 |

CVH: Cardiovascular health; CMM: cardiometabolic multimorbidity; TyG: triglyceride-glucose; CRP:C-reactive protein; Models were fully adjusted for age, sex, race, Townsend deprivation index, education levels, annual household income, family history of heart diseases, history use of lipid lowering drugs, antihypertensives, alcohol intake frequency. The coefficients (β), standard errors (SE), z-values, and corresponding p-values are reported for each term.


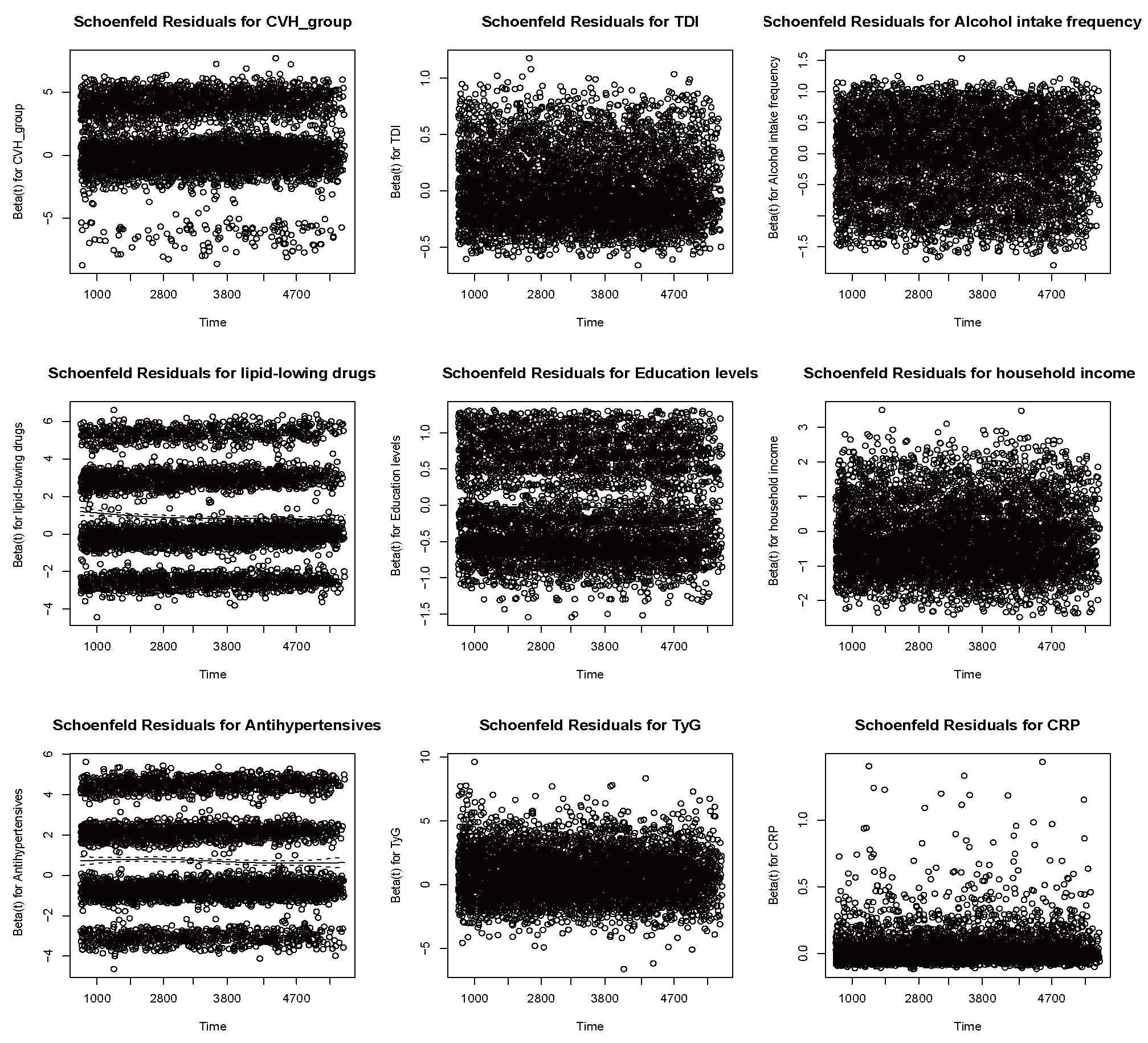


Figure S1. Schoenfeld residual plots for assessing proportional hazards assumption.

CVH: Cardiovascular health; TDI: Townsend deprivation index; TyG: triglyceride-glucose; CRP: C-reactive protein.


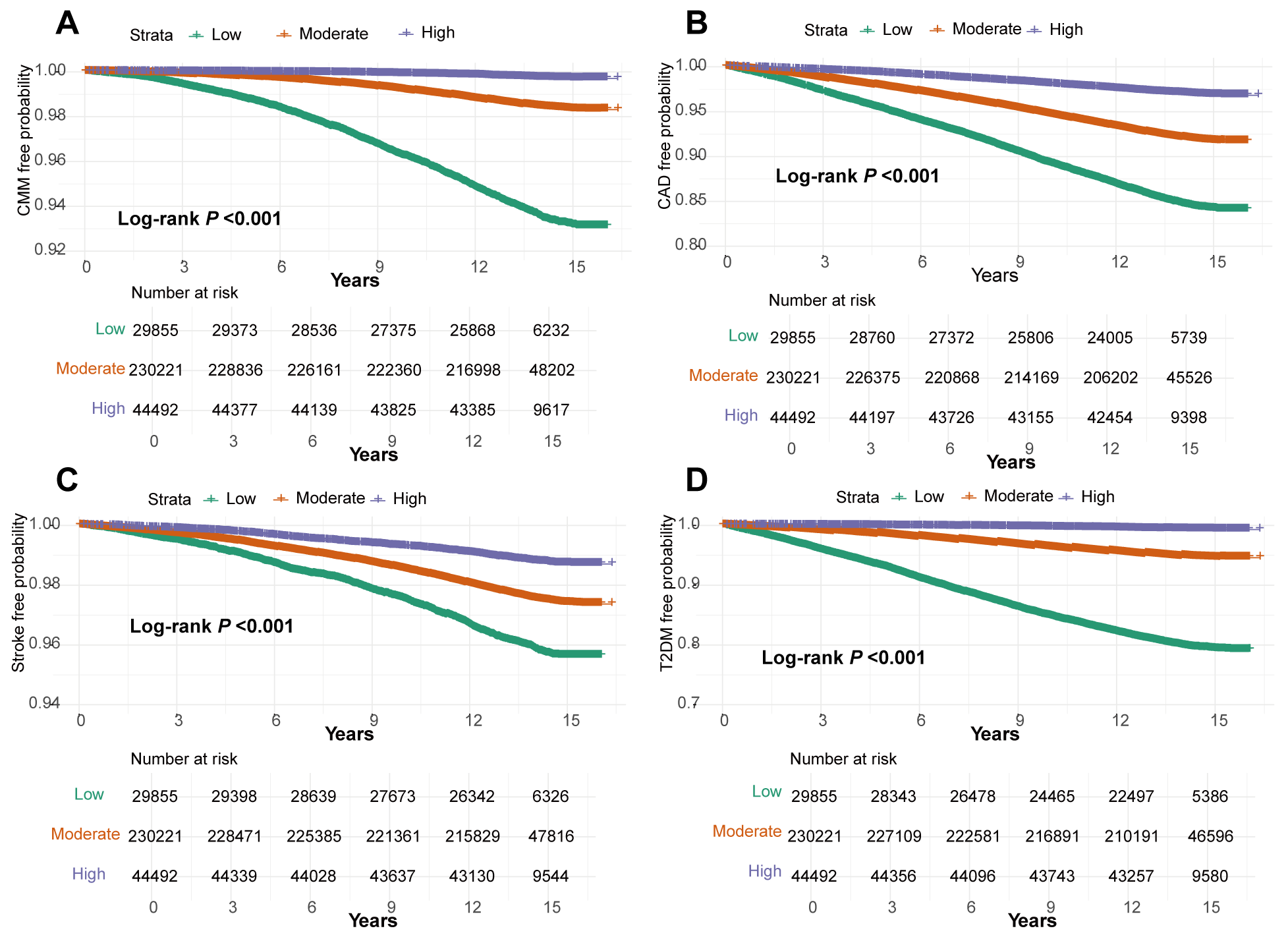


Figure S2. Kaplan–Meier survival curves illustrating the association between CVH groups and the risk of CMM (A), CAD (B), Stroke (C) and T2DM (D).

CVH: Cardiovascular health; CMM: cardiometabolic multimorbidity; CAD: coronary artery disease; T2DM: type 2 diabetes mellitus; Low CVH: LE8 score < 50 points. Moderate CVH: LE8 score 50-80 points. High CVH: LE8 score ≥ 80 points.


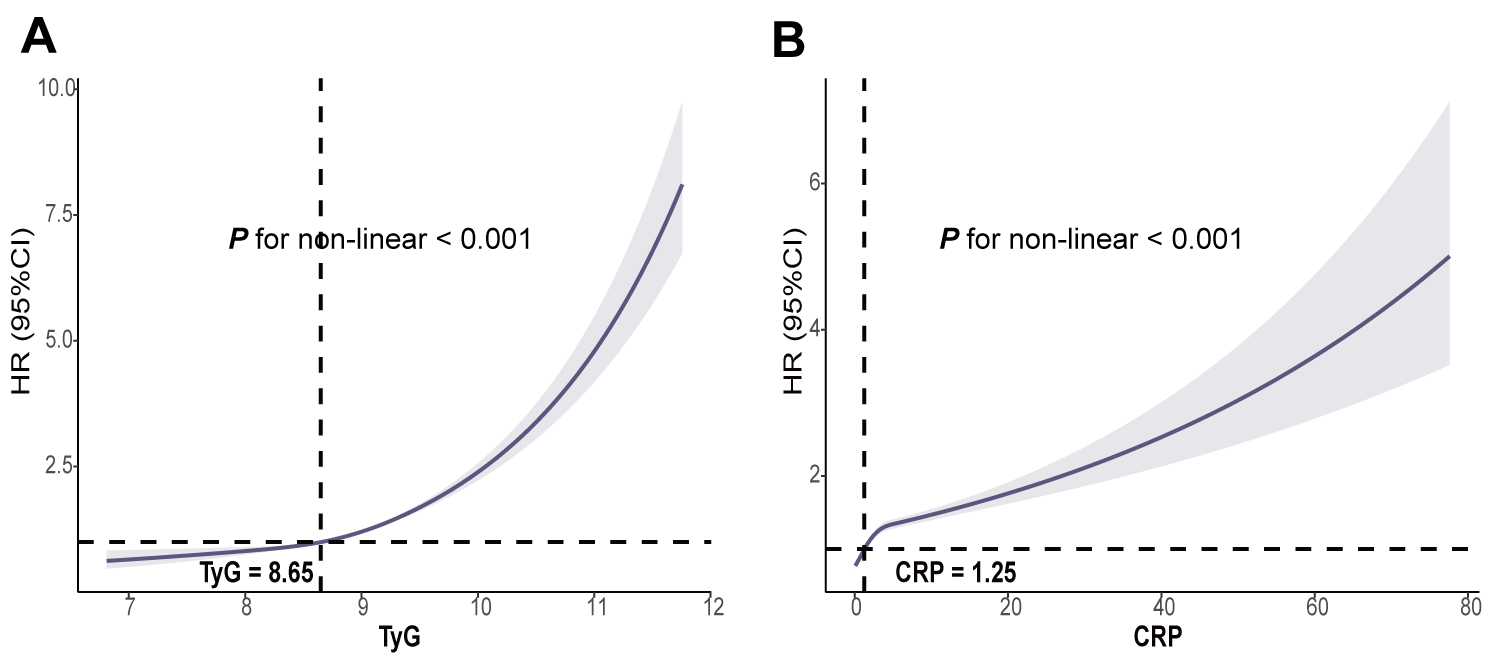


Figure S3. Associations between TyG (A), CRP (B) with CMM were evaluated by RCS.

TyG: triglyceride-glucose; CRP:C-reactive protein; CMM: cardiometabolic multimorbidity; RCS: Restricted cubic spline; HR: hazard ratio; CI: confidence interval. Models were fully adjusted for age, sex, race, Townsend deprivation index, education levels, annual household income, family history of CVD, history use of lipid lowering drugs, antihypertensives, alcohol intake frequency, CRP (for A) and TyG (B).

s.


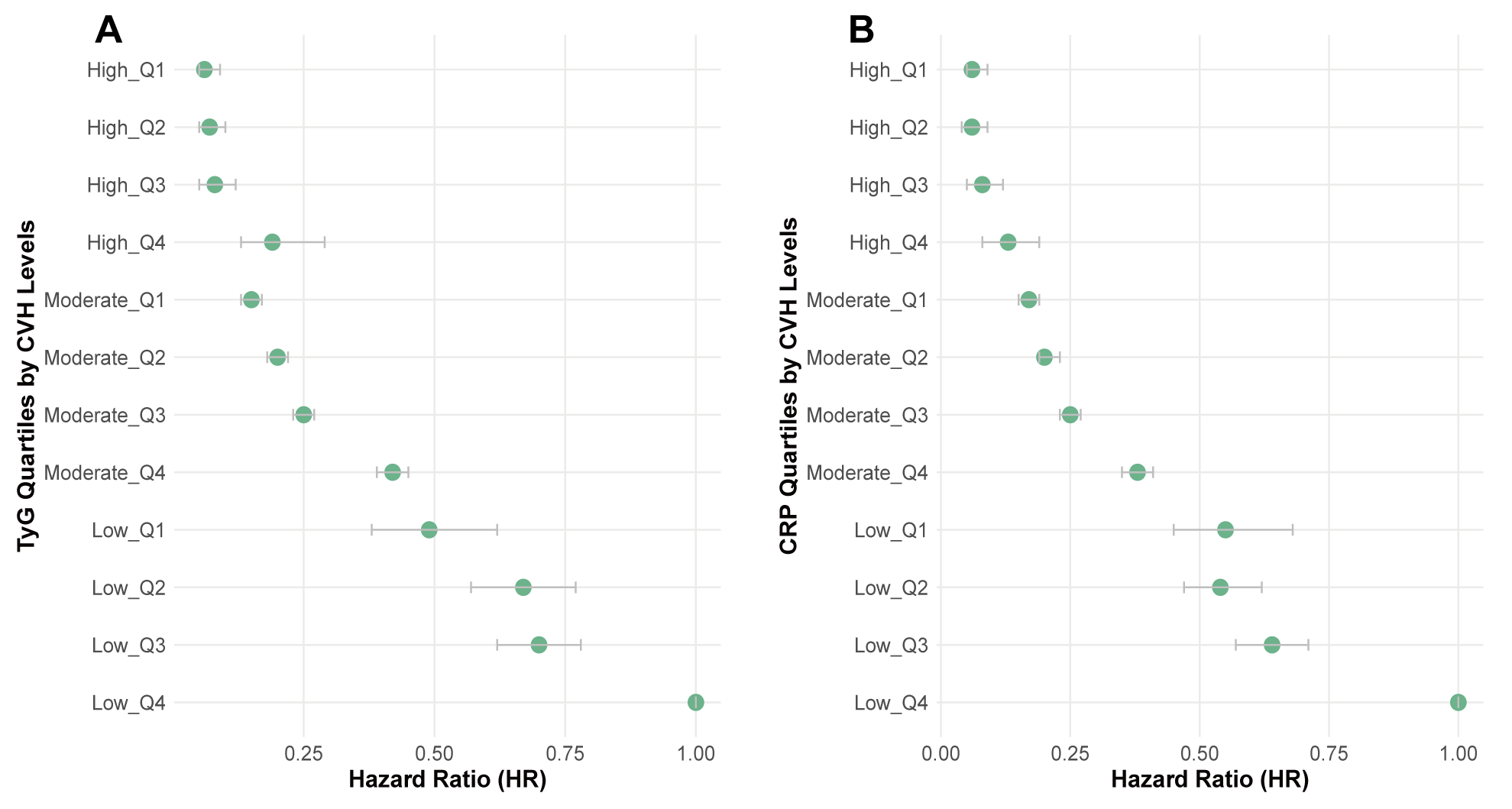


Figure S4. Combined effects of CVH, TyG or CRP and the risk of CMM.

1. Combined effects of CVH and TyG. B. Combined effects of CVH and CRP.

CVH: Cardiovascular health; TyG: triglyceride-glucose; CRP:C-reactive protein; CMM: cardiometabolic multimorbidity; Models were further adjusted for age, sex, race, Townsend deprivation index, education levels, annual household income, family history of heart diseases, history use of lipid lowering drugs, antihypertensives, alcohol intake frequency, CRP (for A) and TyG (B).

**
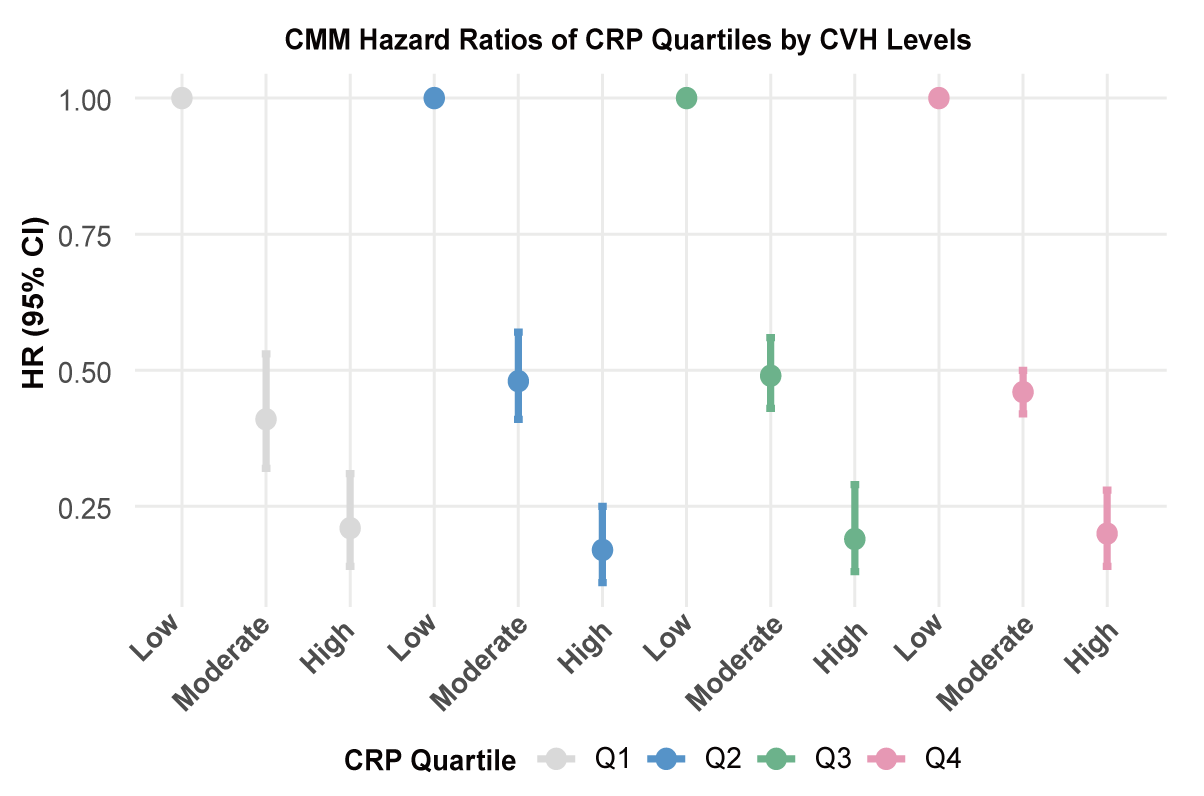
**

Figure S5. Associations between CVH groups with the risk of CMM by CRP quartiles.

CVH: Cardiovascular health; CRP:C-reactive protein; CMM: cardiometabolic multimorbidity; Models were further adjusted for age, sex, race, Townsend deprivation index, education levels, annual household income, family history of heart diseases, history use of lipid lowering drugs, antihypertensives, alcohol intake frequency and TyG.


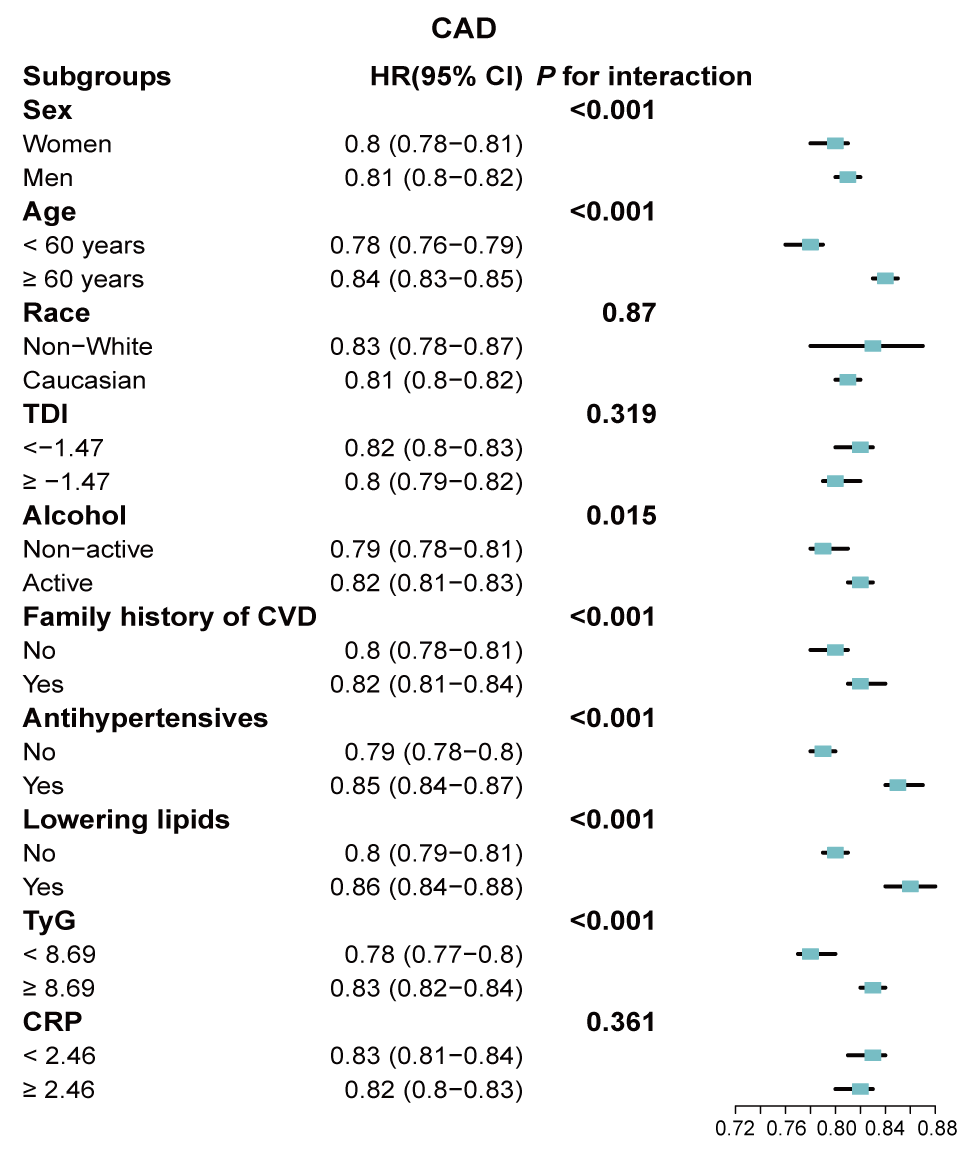


Figure S6. Association between a 10-point increase in LE8 score and the risk of CAD across various subgroups.

LE8: Life’s Essential 8; CAD: coronary artery disease; TyG: triglyceride-glucose; CRP:C-reactive protein; TDI: Townsend deprivation index; HR: hazard ratio; CI: confidence interval. Models were fully adjusted for age, sex, race, TDI, education levels, annual household income, family history of CVD, history use of lipid lowering drugs, antihypertensives, alcohol intake frequency, TyG index and CRP levels.


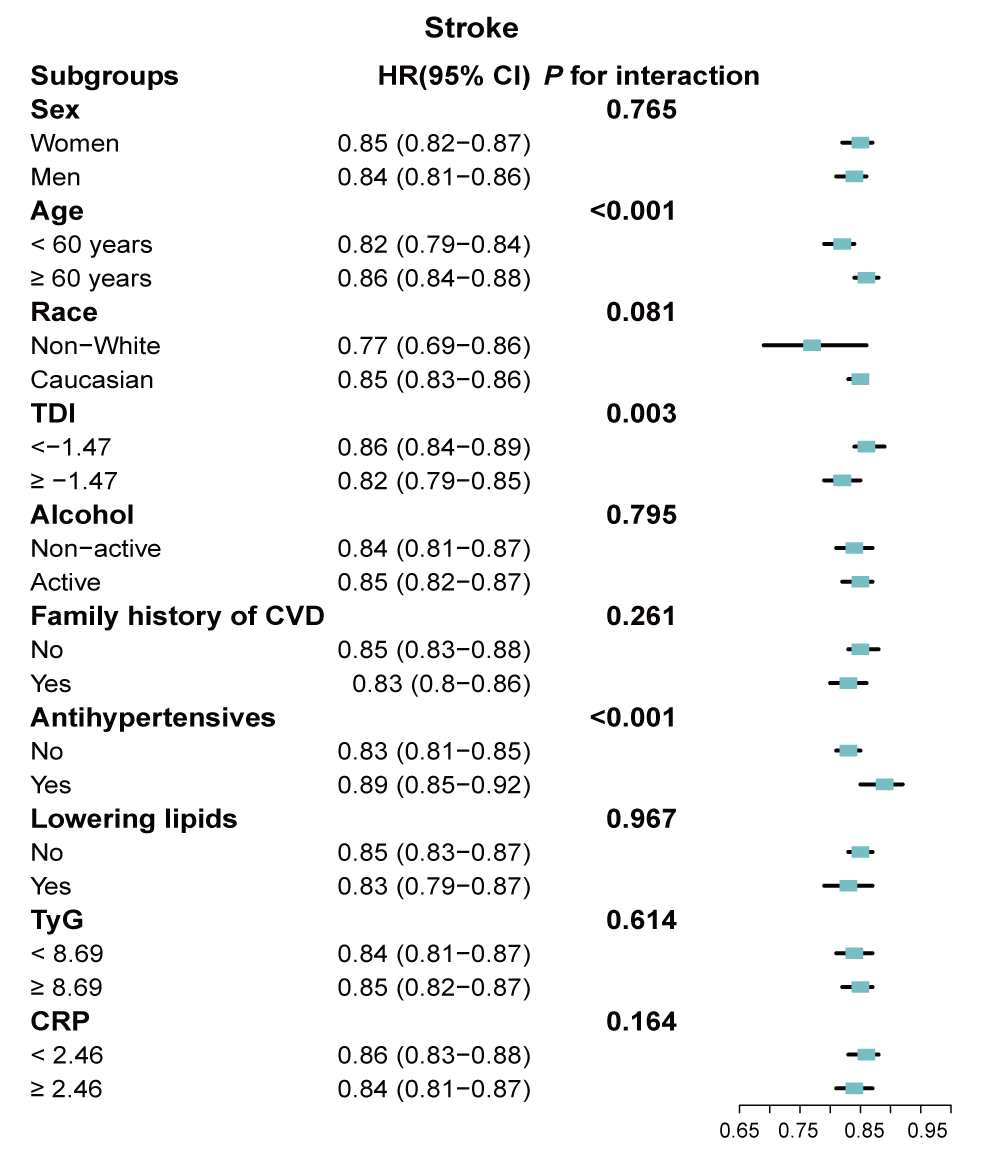


Figure S7. Association between a 10-point increase in LE8 score and the risk of Stroke across various subgroups.

LE8: Life’s Essential 8; TyG: triglyceride-glucose; CRP:C-reactive protein; TDI: Townsend deprivation index; HR: hazard ratio; CI: confidence interval. Models were fully adjusted for age, sex, race, TDI, education levels, annual household income, family history of CVD, history use of lipid lowering drugs, antihypertensives, alcohol intake frequency, TyG index and CRP levels.


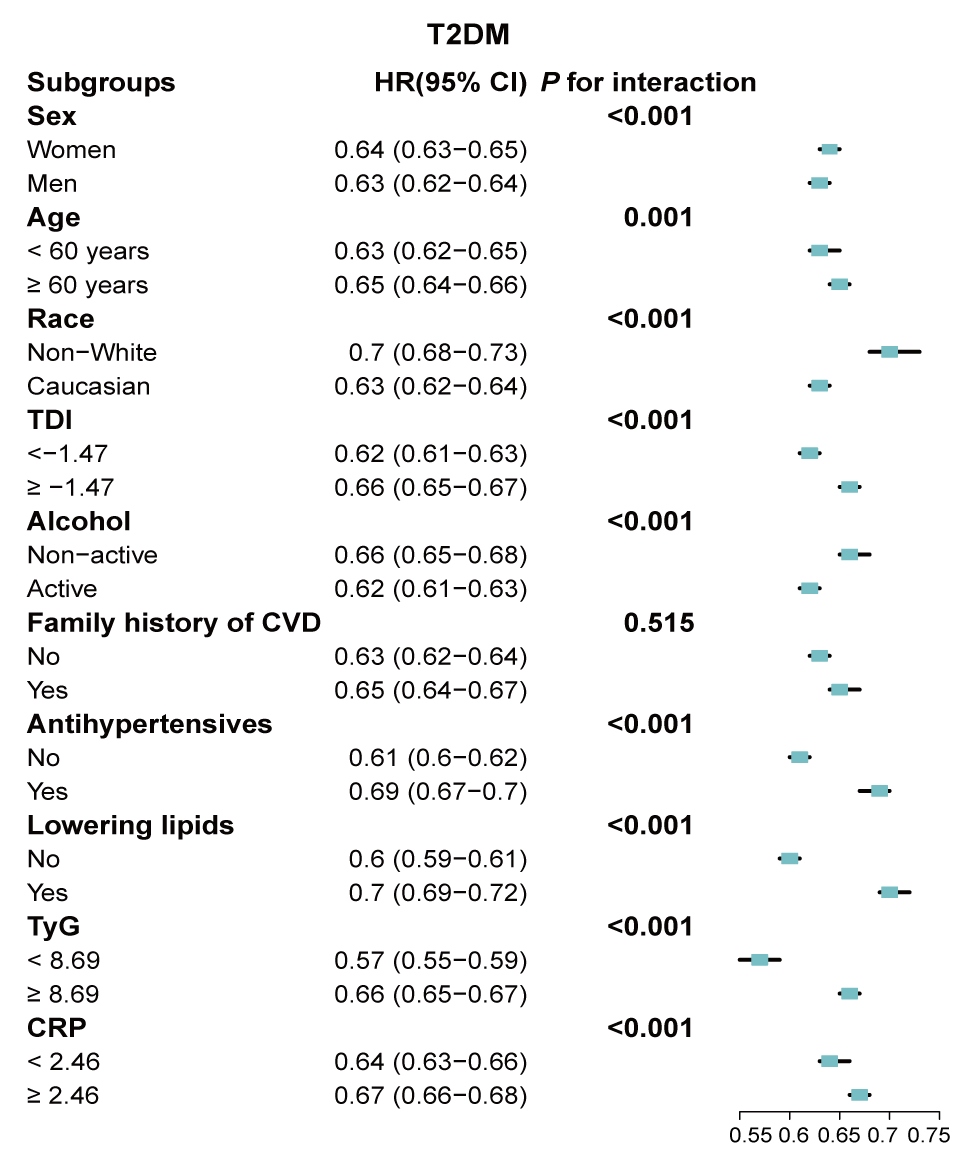


Figure S8. Association between a 10-point increase in LE8 score and the risk of T2DM across various subgroups.

LE8: Life’s Essential 8; T2DM: type 2 diabetes mellitusl; TyG: triglyceride-glucose; CRP:C-reactive protein; TDI: Townsend deprivation index; HR: hazard ratio; CI: confidence interval. Models were fully adjusted for age, sex, race, TDI, education levels, annual household income, family history of CVD, history use of lipid lowering drugs, antihypertensives, alcohol intake frequency, TyG index and CRP levels.


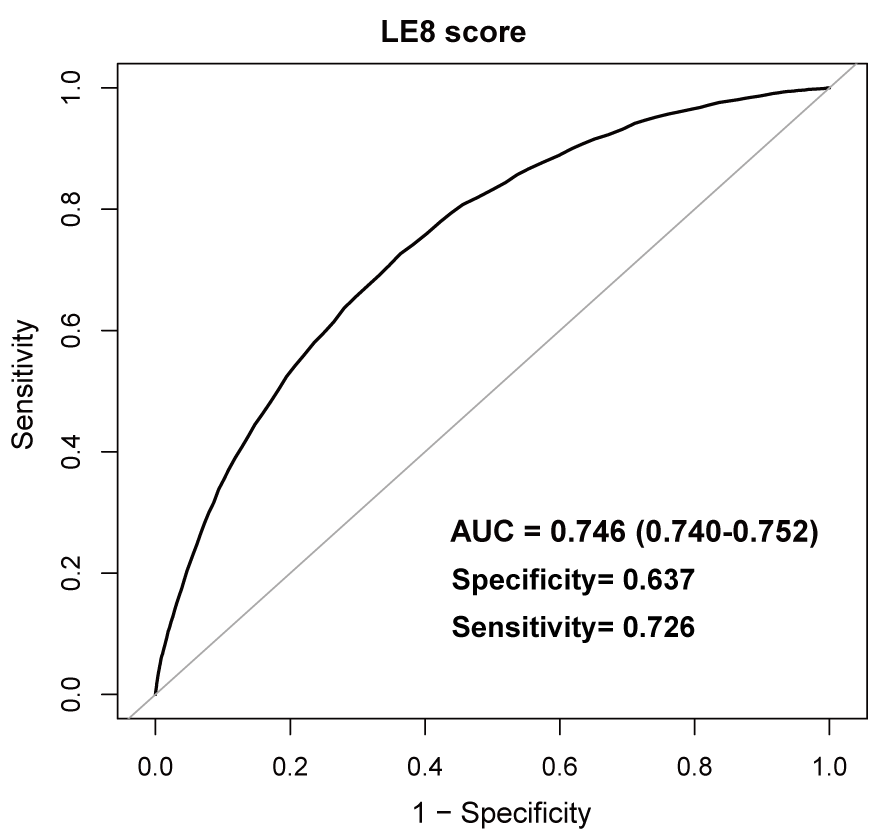


Figure S9. The discriminatory power of LE8 score for the development of CMM.

LE8: Life’s Essential 8; CMM: cardiometabolic multimorbidity;


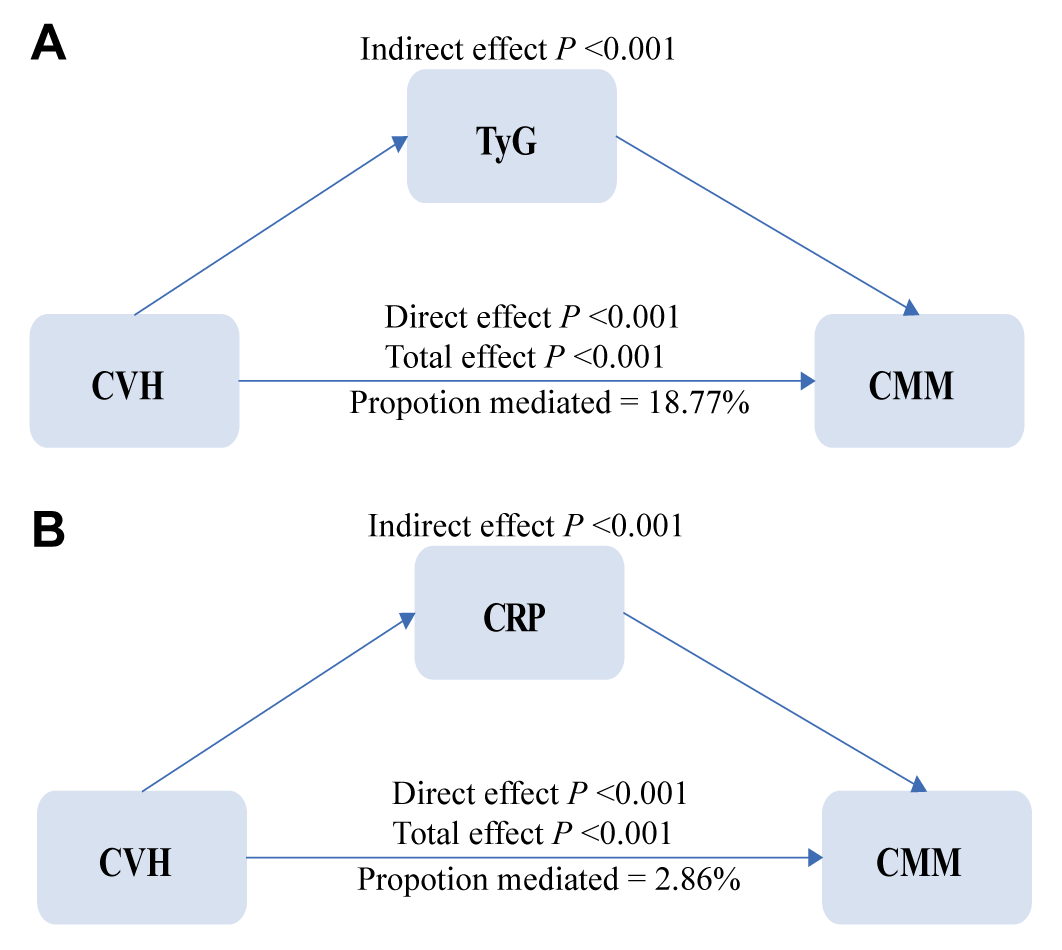


Figure S10. Mediation effect to TyG (A) or CRP (B) on the relationship between LE8 score and the risk of CMM.

LE8: Life’s Essential 8; TyG: triglyceride-glucose; CRP:C-reactive protein; CMM: cardiometabolic multimorbidity; Models were further adjusted for age, sex, race, Townsend deprivation index, education levels, annual household income, family history of heart diseases, history use of lipid lowering drugs, antihypertensives, alcohol intake frequency, CRP (for A) or TyG (B).

Appendix

STROBE Statement—checklist of items that should be included in reports of observational studies

|  | Item No | Recommendation | Page number |
| --- | --- | --- | --- |
| **Title and abstract** | 1 | (*a*) Indicate the study’s design with a commonly used term in the title or the abstract | 2 |
|  |  | (*b*) Provide in the abstract an informative and balanced summary of what was done and what was found | 2 |
| Introduction | | | 3 |
| Background/rationale | 2 | Explain the scientific background and rationale for the investigation being reported | 3 |
| Objectives | 3 | State specific objectives, including any prespecified hypotheses | 3 |
| Methods | | | 3-8 |
| Study design | 4 | Present key elements of study design early in the paper | 4-7 |
| Setting | 5 | Describe the setting, locations, and relevant dates, including periods of recruitment, exposure, follow-up, and data collection | 4-6 |
| Participants | 6 | (*a*) *Cohort study*—Give the eligibility criteria, and the sources and methods of selection of participants. Describe methods of follow-up  *Case-control study*—Give the eligibility criteria, and the sources and methods of case ascertainment and control selection. Give the rationale for the choice of cases and controls  *Cross-sectional study*—Give the eligibility criteria, and the sources and methods of selection of participants | 5-6 |
|  |  | (*b*) *Cohort study*—For matched studies, give matching criteria and number of exposed and unexposed  *Case-control study*—For matched studies, give matching criteria and the number of controls per case |  |
| Variables | 7 | Clearly define all outcomes, exposures, predictors, potential confounders, and effect modifiers. Give diagnostic criteria, if applicable | 5-6 |
| Data sources/ measurement | 8* | For each variable of interest, give sources of data and details of methods of assessment (measurement). Describe comparability of assessment methods if there is more than one group | 4-6 |
| Bias | 9 | Describe any efforts to address potential sources of bias | 6 |
| Study size | 10 | Explain how the study size was arrived at | 6 |
| Quantitative variables | 11 | Explain how quantitative variables were handled in the analyses. If applicable, describe which groupings were chosen and why | 6 |
| Statistical methods | 12 | (*a*) Describe all statistical methods, including those used to control for confounding | 6-8 |
|  |  | (*b*) Describe any methods used to examine subgroups and interactions | 6-8 |
|  |  | (*c*) Explain how missing data were addressed | 6 |
|  |  | (*d*) *Cohort study*—If applicable, explain how loss to follow-up was addressed  *Case-control study*—If applicable, explain how matching of cases and controls was addressed  *Cross-sectional study*—If applicable, describe analytical methods taking account of sampling strategy | 6 |
|  |  | (*e*) Describe any sensitivity analyses |  |

Continued on next page

| Results | | | 8-11 |
| --- | --- | --- | --- |
| Participants | 13* | (a) Report numbers of individuals at each stage of study—eg numbers potentially eligible, examined for eligibility, confirmed eligible, included in the study, completing follow-up, and analysed | 8 |
|  |  | (b) Give reasons for non-participation at each stage |  |
|  |  | (c) Consider use of a flow diagram |  |
| Descriptive data | 14* | (a) Give characteristics of study participants (eg demographic, clinical, social) and information on exposures and potential confounders | 8-9 |
|  |  | (b) Indicate number of participants with missing data for each variable of interest | 8-9 |
|  |  | (c) *Cohort study*—Summarise follow-up time (eg, average and total amount) | 8-9 |
| Outcome data | 15* | *Cohort study*—Report numbers of outcome events or summary measures over time | 8-9 |
|  |  | *Case-control study—*Report numbers in each exposure category, or summary measures of exposure |  |
|  |  | *Cross-sectional study—*Report numbers of outcome events or summary measures |  |
| Main results | 16 | (*a*) Give unadjusted estimates and, if applicable, confounder-adjusted estimates and their precision (eg, 95% confidence interval). Make clear which confounders were adjusted for and why they were included | 9 |
|  |  | (*b*) Report category boundaries when continuous variables were categorized |  |
|  |  | (*c*) If relevant, consider translating estimates of relative risk into absolute risk for a meaningful time period |  |
| Other analyses | 17 | Report other analyses done—eg analyses of subgroups and interactions, and sensitivity analyses | 9-10 |
| Discussion | | | 11-14 |
| Key results | 18 | Summarise key results with reference to study objectives | 11 |
| Limitations | 19 | Discuss limitations of the study, taking into account sources of potential bias or imprecision. Discuss both direction and magnitude of any potential bias | 13-14 |
| Interpretation | 20 | Give a cautious overall interpretation of results considering objectives, limitations, multiplicity of analyses, results from similar studies, and other relevant evidence | 11-14 |
| Generalisability | 21 | Discuss the generalisability (external validity) of the study results | 13-14 |
| Other information | | |  |
| Funding | 22 | Give the source of funding and the role of the funders for the present study and, if applicable, for the original study on which the present article is based |  |

*Give information separately for cases and controls in case-control studies and, if applicable, for exposed and unexposed groups in cohort and cross-sectional studies.

**Note:** An Explanation and Elaboration article discusses each checklist item and gives methodological background and published examples of transparent reporting. The STROBE checklist is best used in conjunction with this article (freely available on the Web sites of PLoS Medicine at http://www.plosmedicine.org/, Annals of Internal Medicine at http://www.annals.org/, and Epidemiology at http://www.epidem.com/). Information on the STROBE Initiative is available at www.strobe-statement.org.
